# Exact global alignment using A* with chaining seed heuristic and match pruning

**DOI:** 10.1093/bioinformatics/btae032

**Published:** 2024-01-23

**Authors:** Ragnar Groot Koerkamp, Pesho Ivanov

**Affiliations:** Department of Computer Science, ETH Zurich, Rämistrasse 101, Zurich 8092, Switzerland; Department of Computer Science, ETH Zurich, Rämistrasse 101, Zurich 8092, Switzerland

## Abstract

**Motivation:**

Sequence alignment has been at the core of computational biology for half a century. Still, it is an open problem to design a practical algorithm for exact alignment of a pair of related sequences in linear-like time.

**Results:**

We solve exact global pairwise alignment with respect to edit distance by using the A* shortest path algorithm. In order to efficiently align long sequences with high divergence, we extend the recently proposed *seed heuristic* with *match chaining*, *gap costs*, and *inexact matches*. We additionally integrate the novel *match pruning* technique and diagonal transition to improve the A* search. We prove the correctness of our algorithm, implement it in the A*PA aligner, and justify our extensions intuitively and empirically.

On random sequences of divergence d=4% and length *n*, the empirical runtime of A*PA scales near-linearly with length (best fit $n^{1.06},\;n\leq 10^{7}\;\text{bp}$). A similar scaling remains up to d=12% (best fit $n^{1.24}$, $n\leq 10^{7}\;\text{bp}$). For $n=10^{7}\;\text{bp}$ and d=4%, A*PA reaches >500× speedup compared to the leading exact aligners Edlib and BiWFA. The performance of A*PA is highly influenced by long gaps. On long (n>500kb) ONT reads of a human sample it efficiently aligns sequences with d<10%, leading to 3× median speedup compared to Edlib and BiWFA. When the sequences come from different human samples, A*PA performs 1.7× faster than Edlib and BiWFA.

**Availability and implementation:**

github.com/RagnarGrootKoerkamp/astar-pairwise-aligner.

## 1 Introduction

The problem of aligning one biological sequence to another is known as *global pairwise alignment* (Navarro 2001). Among others, it is applied to genome assembly, read mapping, variant detection, and multiple sequence alignment (Prjibelski *et al.* 2019). Despite the centrality and age of pairwise alignment (Needleman and Wunsch 1970), ‘a major open problem is to implement an algorithm with linear-like empirical scaling on inputs where the edit distance is linear in *n*’ (Medvedev 2023a).

Alignment accuracy affects subsequent analyses, so a common goal is to find a shortest sequence of edit operations (single-letter insertions, deletions, and substitutions) that transforms one sequence into the other. The length of such a sequence is known as *Levenshtein distance* (Levenshtein 1966) and *edit distance*. It has recently been proven that edit distance cannot be computed in strongly subquadratic time, unless SETH is false (Backurs and Indyk 2015). When the number of sequencing errors is proportional to the length, existing exact aligners scale quadratically both in the theoretical worst case and in practice. Given the increasing amounts of biological data and increasing read lengths, this is a computational bottleneck (Kucherov 2019).

We solve the global alignment problem provably correct and empirically fast by using A* on the alignment graph and building on many existing techniques. Our implementation A*PA (A* Pairwise Aligner) scales near-linear with length up to $10^{7}\;\text{bp}$ long sequences with divergence up to 12%. Additionally, it shows a speedup over other highly optimized aligners when aligning long ONT reads.

### 1.1 Overview of method

To align two sequences *A* and *B* globally with minimal cost, we use the A* shortest path algorithm from the start to the end of the alignment graph, as first suggested by Hadlock (1988a). A core part of the A* algorithm is the heuristic function *h*(*u*) that provides a lower bound on the remaining distance from the current vertex *u*. A good heuristic efficiently computes an accurate estimate *h*, so suboptimal paths get penalized more and A* prioritizes vertices on a shortest path, thus reaching the target quicker. In this article, we extend the *seed heuristic* by Ivanov *et al.* (2022) in several ways to increase its accuracy for long and erroneous sequences.

#### 1.1.1 Seed heuristic (SH)

To define the *seed heuristic* (SH) $h_{s}$, we split *A* into short, non-overlapping substrings (*seeds*) of fixed length *k* (Fig. 2a). Since the whole sequence *A* has to be aligned, each of the seeds also has to be aligned somewhere in *B*. If a seed does not match anywhere in *B* without mistakes, then at least one edit has to be made to align it. Thus, the SH $h_{s}$ is the number of remaining seeds (contained in $A_{\geq i}$) that do not match anywhere in *B*. The SH is a lower bound on the distance between the remaining suffixes $A_{\geq i}$ and $B_{\geq j}$. In order to compute $h_{s}$ efficiently, we precompute all *matches* in *B* for all seeds from *A*. Where Ivanov *et al.* (2022) uses *crumbs* to mark upcoming matches in the graph, we do not need them due to the simpler structure of sequence-to-sequence alignment.

#### 1.1.2 Chaining seed heuristic (CSH)

One drawback of the SH is that it may use matches that do not lie together on a path from *u* to the end, e.g. the matches for *s*_1_ and *s*_3_ in (Fig. 2a). In the *chaining seed heuristic* (CSH) $h_{\text{cs}}$ (Section 3.1), we enforce that the matches occur in the same order in *B* as their corresponding seeds occur in *A*, i.e. the matches form a *chain* going down and right (Fig. 2b). Now, the number of upcoming errors is at least the minimal number of remaining seeds that cannot be aligned on a single chain to the target. When there are many spurious matches (i.e. outside the optimal alignment), chaining improves the accuracy of the heuristic, thus reducing the number of states expanded by A*. To compute CSH efficiently, we subtract the maximal number of matches in a chain starting in the current state from the number of remaining seeds.

#### 1.1.3 Gap-chaining seed heuristic (GCSH)

The CSH penalizes the chaining of two matches by the *seed cost*, the number of skipped seeds in between them. This chaining may skip a different number of letters in *A* and *B*, in which case the absolute difference between these lengths (*gap cost*) is a lower bound on the length of a path between the two matches. The *gap-chaining seed heuristic* (GCSH) $h_{\text{gcs}}$ (Fig. 2c) takes the maximum of the gap cost and the seed cost, which significantly improves the accuracy of the heuristic for sequences with long indels.

#### 1.1.4 Inexact matches

To further improve the accuracy of the heuristic for divergent sequences, we use *inexact matches* (Wu and Manber 1992, Marco-Sola *et al.* 2012). For each seed in *A*, our algorithm now finds all its inexact matches in *B* with cost at most 1. The lack of a match of a seed then implies that at least r=2 edits are needed to align it. This doubles the *potential* of our heuristic to penalize errors.

#### 1.1.5 Match pruning

**Figure 1.**
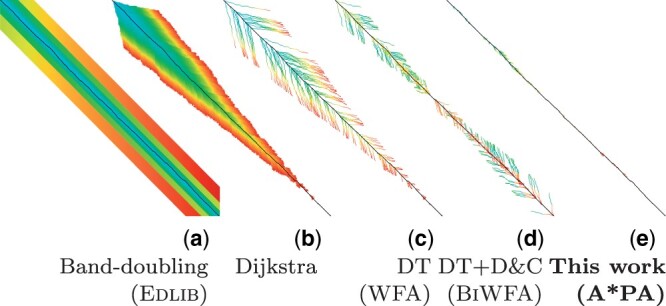
**Computed states per algorithm.** Various optimal alignment algorithms and their implementation are demonstrated on synthetic data (length n = 500 bp, divergence d =16%). The colour indicates the order of computation from blue to red. (a) Band-doubling (Edlib), (b) Dijkstra, (c) Diagonal transition/DT (WFA), (d) DT with divide-andconquer/D&C (BiWFA), (e) A*PA with gap-chaining seed heuristic (GCSH), match pruning, and DT (seed length k = 5 and exact matches).

**Figure 2.**
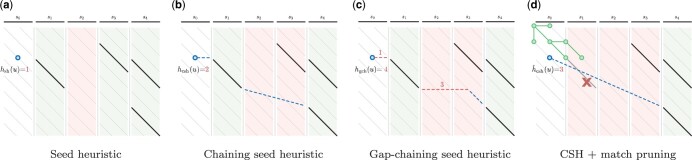
Demonstration of SH, CSH, GCSH, and match pruning. Sequence *A* on top is split into five seeds (horizontal black segments _). Each seed is exactly matched in *B* (diagonal black segments \). The heuristic is evaluated at state *u* (blue circles 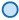), based on the four remaining seeds. The heuristic value is based on a maximal chain of matches (green columns 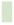 for seeds with matches; red columns 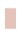 otherwise). Dashed lines denote chaining of matches. (a) The SH $h_{s}(u)=1$ is the number of remaining seeds that do not have matches (only *s*_2_). (b) The CSH $h_{\text{cs}}(u)=2$ is the number of remaining seeds without a match (*s*_2_ and *s*_3_) on a path going only down and to the right containing a maximal number of matches. (c) The GCSH $h_{\text{gcs}}(u)=4$ is minimal cost of a chain, where the cost of joining two matches is the maximum of the number of not matched seeds and the gap cost between them. Red dashed lines denote gap costs. (d) Once the start or end of a match is expanded (green circles 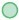), the match is *pruned* (red cross 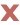), and future computations of the heuristic ignore it. *s*_1_ is removed from the maximum chain of matches starting at *u* so ${\hat{h}}_{\text{cs}}(u)$ increases by 1.

In order to further improve the accuracy of our heuristic, we apply the *multiple-path pruning* observation (Poole and Mackworth 2017): once a shortest path to a vertex *u* has been found, no other path to *u* can be shorter. Since we search for a single shortest path, we want to incrementally update our heuristic (similar to Real-Time Adaptive A* (Koenig and Likhachev 2006)) to penalize further paths to *u*. We prove that once A* expands a state *u*, which is at the start or end of a match, indeed it has found a shortest path to *u*. Then, we can ignore (*prune*) such a match, thus penalizing other paths to *u* (Fig 2d and Section 3.2). Pruning increases the heuristic in states preceding the match, thereby penalizing states preceding the ‘tip’ of the A* search. This reduces the number of expanded states, and leads to near-linear scaling with sequence length (Fig. 1e).

#### 1.1.6 Diagonal transition

The diagonal-transition algorithm only visits so called *farthest-reaching* states (Ukkonen 1985, Myers 1986) along each diagonal and lies at the core of wavefront alignment (WFA) algorithm (Marco-Sola *et al.* 2021) (Fig. 1c). We introduce the *diagonal-transition* optimization to the A* algorithm that skips states known to be not farthest reaching. This is independent of the A* heuristic and makes the exploration more ‘hollow’, especially speeding up the quadratic behaviour of A* in complex regions.

We present an algorithm to efficiently initialize and evaluate these heuristics and optimizations (Supplementary Section A and Section 3.3), prove the correctness of our methods (Supplementary Section B), and evaluate and compare their performance to other optimal aligners (Section 4 and Supplementary Section C).

### 1.2 Related work

We first outline the algorithms behind the fastest exact global aligners: dynamic programming (DP)-based band-doubling (used by Edlib) and diagonal transition (DT) (used by BiWFA). Then, we outline methods that A*PA integrates.

#### 1.2.1 Dynamic programming

This classic approach to aligning two sequences computes a table where each cell contains the edit distance between a prefix of the first sequence and a prefix of the second by reusing the solutions for shorter prefixes. This quadratic DP was introduced for speech signals Vintsyuk (1968) and genetic sequences (Needleman and Wunsch 1970, Sankoff 1972, Sellers 1974, Wagner and Fischer 1974). The quadratic *O*(*nm*) runtime for sequences of lengths *n* and *m* allowed for aligning of long sequences for the time but speeding it up has been a central goal in later works. Implementations of this algorithm include SeqAn (Reinert *et al.* 2017) and Parasail (Daily 2016).

#### 1.2.2 Band-doubling and bit-parallelization

When the aligned sequences are similar, the whole DP table does not need to be computed. One such output-sensitive algorithm is the *band-doubling* algorithm of Ukkonen (1985) (Fig. 1a), which considers only states around the main diagonal of the table, in a *band* with exponentially increasing width, leading to *O*(*ns*) runtime, where *s* is the edit distance between the sequences. This algorithm, combined with the *bit-parallel optimization* by Myers (1999) is implemented in Edlib (Šošić and Šikić 2017) with O(ns/w) runtime, where *w* is the machine word size (nowadays 64).

#### 1.2.3 Diagonal transition

*DT* (Ukkonen 1985, Myers 1986) is a technique, which exploits the observation that the edit distance does not decrease along diagonals of the DP matrix. This allows for an equivalent representation of the DP table based on *farthest-reaching states* for a given edit distance along each diagonal. DT has an *O*(*ns*) worst-case runtime but only takes expected $O(n+s^{2})$ time (Fig. 1c) for random input sequences (Myers 1986) (which is still quadratic for a fixed divergence d=s/n). It has been extended to linear and affine costs in the WFA (Marco-Sola *et al.* 2021) in a way similar to Gotoh (1982). Its memory usage has been improved to linear in BiWFA (Marco-Sola *et al.* 2023) by combining it with the divide-and-conquer approach of Hirschberg (1975), similar to Myers (1986) for unit edit costs. Wu *et al.* (1990) and Papamichail and Papamichail (2009) apply DT to align sequences of different lengths.

#### 1.2.4 Contours

**Figure 3.**
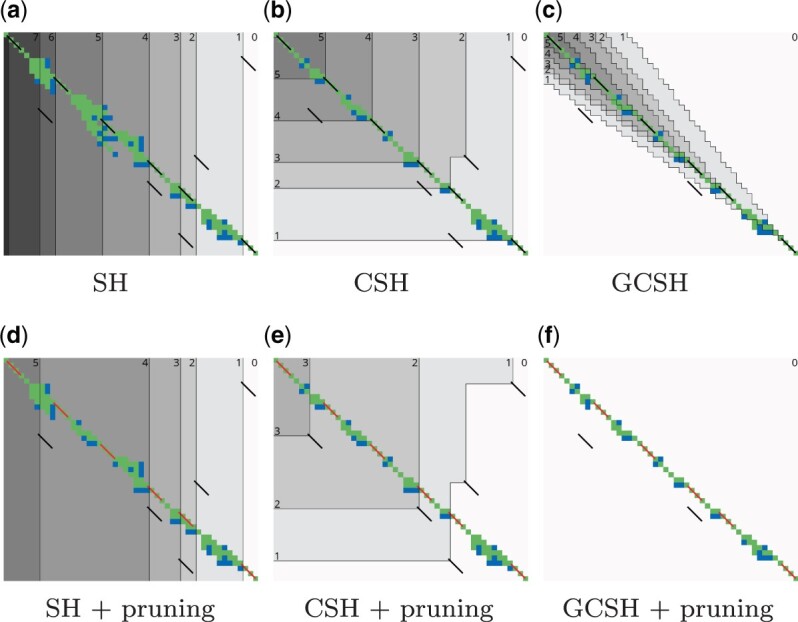
Contours and layers of different heuristics after aligning (n=48, m=42, r=1, k=3, edit distance 10). Exact matches are black diagonal segments (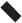). The background colour indicates $S_{p}(u)$, the maximum number of matches on a ${⪯}_{p}$-chain from *u* to the end starting, with $S_{p}(u)=0$ in white. The thin black boundaries of these regions are *Contours*. The states of layer $L_{\ell }$*precede* contour ℓ. Expanded states are green (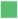), open states blue (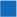), and pruned matches red (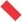). Pruning matches changes the contours and layers. GCSH ignores matches m⪯Tvt.

The longest common subsequence (LCS) problem is a special case of edit distance, in which gaps are allowed but substitutions are forbidden. *Contours* partition the state-space into regions with the same remaining answer of the LCS subtask (Fig. 3). The contours can be computed in log-linear time in the number of matching elements between the two sequences, which is practical for large alphabets (Hirschberg 1977, Hunt and Szymanski 1977).

#### 1.2.5 Shortest paths and A*

An alignment that minimizes edit distance corresponds to a shortest path in the *alignment graph* (Vintsyuk 1968, Ukkonen 1985). Assuming non-negative edit costs, a shortest path can be found using Dijkstra’s algorithm (Ukkonen 1985) (Fig. 1b) or A* (Hart *et al.* 1968). A* is an informed search algorithm, which uses a task-specific heuristic function to direct its search, and has previously been applied to the alignment graph by Hadlock (1988a, b) and Spouge (1989, 1991). A* with an accurate heuristic may find a shortest path significantly faster than an uninformed search, such as Dijkstra’s algorithm.

#### 1.2.6 A* heuristics

One widely used heuristic function is the *gap cost* that counts the minimal number of indels needed to align the suffixes of two sequences (Ukkonen 1985, Spouge 1989, Wu *et al.* 1990, Myers and Miller 1995, Papamichail and Papamichail 2009, Šošić and Šikić 2017). Hadlock (1988a) introduces a heuristic based on character frequencies.

#### 1.2.7 Seed-and-extend

*Seed-and-extend* is a commonly used paradigm for approximately solving semi-global alignment by first matching similar regions between sequences (*seeding*) to find *matches* (also called *anchors*), followed by *extending* these matches (Kucherov 2019). Aligning long reads requires the additional step of chaining the seed matches (*seed-chain-extend*). Seeds have also been used to solve the LCSk generalization of LCS (Benson *et al.* 2013, Pavetić *et al.* 2017). Except for the SH (Ivanov *et al.* 2022), most seeding approaches seek for seeds with accurate long matches.

#### 1.2.8 Seed heuristic

A* with *SH* is an exact algorithm that was recently introduced for exact semi-global sequence-to-graph alignment (Ivanov *et al.* 2022). In a precomputation step, the query sequence is split into non-overlapping *seeds* each of which is matched exactly to the reference. When A* explores a new state, the SH is computed as the number of remaining seeds that cannot be matched in the upcoming reference. A* with the SH enables provably exact alignment but runs reasonably fast only when the long sequences are very similar (≤0.3% divergence).

### 1.3 Contributions

We present an algorithm for exact global alignment that uses A* on the alignment graph (Hart *et al.* 1968, Hadlock 1988a), starting with the SH of Ivanov *et al.* (2022).

We increase the accuracy of this heuristic in several novel ways: seeds must match in order in the *CSH*, and gaps between seeds are penalized in the *GCSH*. The novel *match pruning* technique penalizes states ‘lagging behind’ the tip of the search and turns the otherwise quadratic algorithm into an empirically near-linear algorithm in many cases. Inexact matches (Wu and Manber 1992, Marco-Sola *et al.* 2012) increase the divergence of sequences that can be efficiently aligned. We additionally apply the diagonal-transition algorithm (Ukkonen 1985, Myers 1986), so that only the small fraction of farthest-reaching states needs to be computed. We prove the correctness of our methods, and apply contours (Hirschberg 1977, Hunt and Szymanski 1977) to efficiently initialize and evaluate the heuristic. We implement our method in the novel aligner A*PA.

On uniform-random synthetic data with 4% divergence, the runtime of A*PA scales linearly with length up to $10^{7}\;\text{bp}$ and is up to 500× faster than Edlib and BiWFA. On >500 kb long Oxford Nanopore Technologies (ONT) reads of the human genome, A*PA is 3× faster in median than Edlib and BiWFA when only read errors are present, and 1.7× faster in median when additionally genetic variation is present.

## 2 Preliminaries

This section provides definitions and notation that are used throughout the article. A summary of notation is included in Supplementary Section D.

### 2.1 Sequences

The input sequences $A=\bar{a_{0}a_{1}\ldots a_{i}\ldots a_{n-1}}$ and $B=\bar{b_{0}b_{1}\ldots b_{j}\ldots b_{m-1}}$ are over an alphabet Σ with four letters. We refer to substrings $\bar{a_{i}\ldots a_{i\prime -1}}$ as $A_{i\ldots i\prime }$, to prefixes $\bar{a_{0}\ldots a_{i-1}}$ as $A_{<i}$, and to suffixes $\bar{a_{i}\ldots a_{n-1}}$ as $A_{\geq i}$. The *edit distance* ed(A,B) is the minimum number of insertions, deletions, and substitutions of single letters needed to convert *A* into *B*. The *divergence* is the observed number of errors per letter, d:=ed(A,B)/n, whereas the *error rate e* is the number of errors per letter *applied* to a sequence.

### 2.2 Alignment graph

Let *state* 〈i,j〉 denote the subtask of aligning the prefix $A_{<i}$ to the prefix $B_{<j}$. The *alignment graph* (also called *edit graph*) *G*(*V*, *E*) is a weighted directed graph with vertices V={〈i,j〉 | 0≤i≤n,0≤j≤m} corresponding to all states, and edges connecting subtasks: edge 〈i,j〉→〈i+1,j+1〉 has cost zero if $a_{i}=b_{j}$ (match) and one otherwise (substitution), and edges 〈i,j〉→〈i+1,j〉 (deletion) and 〈i,j〉→〈i,j+1〉 (insertion) have cost 1. We denote the starting state 〈0,0〉 by *v_s_*, the target state 〈n,m〉 by *v_t_*, and the distance between states *u* and *v* by d(u,v). For brevity, we write f〈i,j〉 instead of f(〈i,j〉).

### 2.3 Paths and alignments

A path *π* from 〈i,j〉 to 〈i′,j′〉 in the alignment graph *G* corresponds to a *(pairwise) alignment* of the substrings $A_{i\ldots i\prime }$ and $B_{j\ldots j\prime }$ with cost $c_{\text{path}}(\pi )$. A shortest path $\pi^{*}$ from *v_s_* to *v_t_* corresponds to an optimal alignment, thus, $c_{\text{path}}(\pi^{*})=d(v_{s},v_{t})=\text{ed}(A,B)$. We write $g^{*}(u):=d(v_{s},u)$ for the distance from the start to *u* and $h^{*}(u):=d(u,v_{t})$ for the distance from *u* to the target.

### 2.4 Seeds and matches

We split the sequence *A* into a set of consecutive non-overlapping substrings (*seeds*) $S=\{s_{0},s_{1},s_{2},\ldots ,s_{\left\lfloor n/k\right\rfloor -1}\}$, such that each seed $s_{l}=A_{lk\ldots lk+k}$ has length *k*. After aligning the first *i* letters of *A*, our heuristics will only depend on the *remaining* seeds $S_{\geq i}:=\{s_{l}\in S\;|\;lk\geq i\}$ contained in the suffix $A_{\geq i}$. We denote the set of seeds between u=〈i,j〉 and v=〈i′,j′〉 by $S_{u\ldots v}=S_{i\ldots i\prime }=\{s_{l}\in S\;|\;i\leq lk,lk+k\leq i\prime \}$ and an *alignment* of *s* to a subsequence of *B* by *π_s_*. The alignments of seed *s* with sufficiently low cost (Section 3.1) form the set $M_{s}$ of *matches*.

### 2.5 Dijkstra and A*

Dijkstra’s algorithm (Dijkstra 1959) finds a shortest path from *v_s_* to *v_t_* by *expanding* (generating all successors) vertices in order of increasing distance $g^{*}(u)$ from the start. Each vertex to be expanded is chosen from a set of *open* vertices. The A* algorithm (Hart *et al.* 1968, 1972, Pearl 1984), instead directs the search towards a target by expanding vertices in order of increasing f(u):=g(u)+h(u), where *h*(*u*) is a heuristic function that estimates the distance $h^{*}(u)$ to the end and *g*(*u*) is the shortest length of a path from *v_s_* to *u* found so far. A heuristic is *admissible* if it is a lower bound on the remaining distance, $h(u)\leq h^{*}(u)$, which guarantees that A* has found a shortest path as soon as it expands *v_t_*. Heuristic *h*_1_*dominates* (is *more accurate* than) another heuristic *h*_2_ when $h_{1}(u)\geq h_{2}(u)$ for all vertices *u*. A dominant heuristic will usually, but not always (Holte 2010), expand less vertices. Note that Dijkstra’s algorithm is equivalent to A* using a heuristic that is always 0, and that both algorithms require non-negative edge costs. Our variant of the A* algorithm is provided in Supplementary Section A.1.

### 2.6 Chains

A state u=〈i,j〉∈V*precedes* a state v=〈i′,j′〉∈V, denoted u⪯v, when i≤i′ and j≤j′. Similarly, a match *m* precedes a match m′, denoted m⪯m′, when the end of *m* precedes the start of m′. This makes the set of matches a partially ordered set. A state *u* precedes a match *m*, denoted u⪯m, when it precedes the start of the match. A *chain* of matches is a (possibly empty) sequence of matches $m_{1}⪯\ldots ⪯m_{l}$.

### 2.7 Gap cost

The number of indels to align substrings $A_{i\ldots i\prime }$ and $B_{j\ldots j\prime }$ is at least their difference in length: $c_{\text{gap}}(\langle i,j\rangle ,\langle i\prime ,j\prime \rangle ):=|(i\prime -i)-(j\prime -j)\;|$. For u⪯v⪯w, the gap cost satisfies the triangle inequality $c_{\text{gap}}(u,w)\leq c_{\text{gap}}(u,v)+c_{\text{gap}}(v,w)$.

### 2.8 Contours

To efficiently calculate maximal chains of matches, *contours* are used. Given a set of matches M, *S*(*u*) is the number of matches in the longest chain $u⪯m_{1}⪯\ldots$, starting at *u*. The function S〈i,j〉 is non-increasing in both *i* and *j*. *Contours* are the boundaries between regions of states with S(u)=ℓ and S(u)<ℓ (Fig. 3). Note that contour ℓ is completely determined by the set of matches m∈M for which S(start(m))=ℓ (Hirschberg 1977). Hunt and Szymanski (1977) give an algorithm to efficiently compute *S* when M is the set of single-letter matches between *A* and *B*, and Deorowicz and Grabowski (2014) give an algorithm when M is the set of exact *k*-mer matches.

## 3 Methods

We formally define the general CSH (Section 3.1) that encompases *inexact matches*, *chaining*, and *gap costs* (Fig. 2). Next, we introduce the *match pruning* (Section 3.2) improvement and integrate our A* algorithm with the *diagonal-transition* optimization (Supplementary Section A.2). We present a practical algorithm (Section 3.3), implementation (Supplementary Section A.3), and proofs of correctness (Supplementary Section B).

### 3.1 General CSH

We introduce three heuristics for A* that estimate the edit distance between a pair of suffixes. Each heuristic is an instance of a *general CSH*. After splitting the first sequence into seeds S, and finding all matches M in the second sequence, any shortest path to the target can be partitioned into a *chain* of matches and connections between the matches. Thus, the cost of a path is the sum of match costs $c_{m}$ and *chaining costs γ*. Our simplest SH ignores the position in *B* where seeds match and counts the number of seeds that were not matched ($\gamma =c_{\text{seed}}$). To efficiently handle more errors, we allow seeds to be matched inexactly, require the matches in a path to be ordered (CSH), and include the gap-cost in the chaining cost $\gamma =\max(c_{\text{gap}},c_{\text{seed}})$ to penalize indels between matches (GCSH).

#### 3.1.1 Inexact matches

We generalize the notion of exact matches to *inexact matches*. We fix a threshold cost *r* (0<r≤k) called the *seed potential* and define the set of *matches* $M_{s}$ as all alignments *m* of seed *s* with *match cost* $c_{m}(m)<r$. The inequality is strict so that $M_{s}=\emptyset$ implies that aligning the seed will incur cost at least *r*. Let $M=\underset{s}{\cup }M_{s}$ denote the set of all matches. With r=1, we allow only *exact* matches, while with r=2, we allow both exact and *inexact* matches with one edit. We do not consider higher *r* in this article. For notational convenience, we define $m_{\omega }\notin M$ to be a match from *v_t_* to *v_t_* of cost 0.

#### 3.1.2 Potential of a heuristic

We call the maximal value the heuristic can take in a state its *potential P*. The potential of our heuristics in state 〈i,j〉 is the sum of seed potentials *r* over all seeds after *i*: $P\langle i,j\rangle :=r\cdot \;|\;S_{\geq i}\;|\;$.

#### 3.1.3 Chaining matches

Each heuristic limits how matches can be *chained* based on a *partial order* on states. We write $u\;{⪯}_{p}\;v$ for the partial order implied by a function *p*: p(u)⪯p(v). A ${⪯}_{p}$*-chain* is a sequence of matches $m_{1}\;{⪯}_{p}\;\ldots \;{⪯}_{p}\;m_{l}$ that precede each other: $\text{end}(m_{i})\;{⪯}_{p}\;\text{start}(m_{i+1})$ for 1≤i<l. To chain matches according only to their *i*-coordinate, SH is defined using ${⪯}_{i}$-chains, while CSH and GCSH are defined using ⪯ that compares both *i* and *j*.

#### 3.1.4 Chaining cost

The *chaining cost γ* is a lower bound on the path cost between two consecutive matches: from the end state *u* of a match, to the start *v* of the next match.

For SH and CSH, the *seed cost* is *r* for each seed that is not matched: $c_{\text{seed}}(u,v):=r\cdot |S_{u\ldots v}|$. When $u\;{⪯}_{i}\;v$ and *v* is not in the interior of a seed, then $c_{\text{seed}}(u,v)=P(u)-P(v)$.

For GCSH, we also include the gap cost $c_{\text{gap}}(\langle i,j\rangle ,\langle i\prime ,j\prime \rangle ):=|(i\prime -i)-(j\prime -j)|$, which is the minimal number of indels needed to correct for the difference in length between the substrings $A_{i\ldots i\prime }$ and $B_{j\ldots j\prime }$ between two consecutive matches (Section 2). Combining the seed cost and the chaining cost, we obtain the gap-seed cost $c_{\text{gs}}=\max(c_{\text{seed}},c_{\text{gap}})$, which is capable of penalizing long indels and we use for GCSH. Note that $\gamma =c_{\text{seed}}+c_{\text{gap}}$ would not give an admissible heuristic since indels could be counted twice, in both $c_{\text{seed}}$ and $c_{\text{gap}}$.

For conciseness, we also define *γ*, $c_{\text{seed}},\;c_{\text{gap}}$, and $c_{\text{gs}}$ between matches γ(m,m′):=γ(end(m),start(m′)), from a state to a match γ(u,m′):=γ(u,start(m′)), and from a match to a state γ(m,u)=γ(end(m),u).

#### 3.1.5 General CSH

We define the general CSH used to instantiate SH, CSH, and GCSH.

Definition 1 (General CSH) Given a set of matches M, partial order ${⪯}_{p}$, and chaining cost *γ*, the *general CSH* $h_{p,\gamma }^{M}(u)$ is the minimal sum of match costs and chaining costs over all ${⪯}_{p}$-chains (indexing extends to $m_{0}:=u$ and $m_{l+1}:=m_{\omega }$):

$$h_{p,\gamma }^{M}(u):=\min_{\begin{array}{c} u{⪯}_{p}m_{1}{⪯}_{p}\ldots {⪯}_{p}m_{l}{⪯}_{p}v_{t}\\ m_{i}\in M \end{array}}\sum_{0\leq i\leq l}[\gamma (m_{i},m_{i+1})+c_{m}(m_{i+1})].$$

We instantiate our heuristics according to Table 1. Our admissibility proofs (Supplementary Section B.1) are based on $c_{m}$ and *γ* being lower bounds on disjoint parts of the remaining path. The more complex $h_{\text{gcs}}$ dominates the other heuristics and usually expands fewer states.

**Table 1.** Definitions of our heuristic functions.

Heuristic		Order	Chaining cost *γ*
$h_{s}(u)$	Seed heuristic (SH)	${⪯}_{i}$	$c_{\text{seed}}$
$h_{\text{cs}}(u)$	Chaining seed h. (CSH)	⪯	$c_{\text{seed}}$
$h_{\text{gcs}}(u)$	Gap-chaining seed h. (GCSH)	⪯	$\max(c_{\text{gap}},c_{\text{seed}})$

Notes: SH orders the matches by *i* and uses only the seed cost. CSH orders the matches by both *i* and *j*. GCSH additionally exploits the gap cost.

Theorem 1 *The SH* $h_{s}$*, the CSH* $h_{\text{cs}}$*, and the GCSH* $h_{\text{gcs}}$*are* admissible*. Furthermore*, $h_{s}^{M}(u)\leq h_{\text{cs}}^{M}(u)\leq h_{\text{gcs}}^{M}(u)$*for all states u.*

We are now ready to instantiate A* with our admissible heuristics but we will first improve them and show how to compute them efficiently.

### 3.2 Match pruning

In order to reduce the number of states expanded by the A* algorithm, we apply the *multiple-path pruning* observation: once a shortest path to a state has been found, no other path to this state could possibly improve the global shortest path (Poole and Mackworth 2017). As soon as A* expands the start or end of a match, we *prune* it, so that the heuristic in preceding states no longer benefits from the match, and they get deprioritized by A*. We define *pruned* variants of all our heuristics that ignore pruned matches:

Definition 2 (Pruning heuristic) Let *E* be the set of expanded states during the A* search, and let M\E be the set of matches that were not pruned, i.e. those matches not starting or ending in an expanded state. We say that $\hat{h}:=h^{M\backslash E}$ is a *pruning heuristic* version of *h*.

The hat over the heuristic function ($\hat{h}$) denotes the implicit dependency on the progress of the A*, where at each step a different $h^{M\backslash E}$ is used. Our modified A* algorithm (Supplementary Section A.1) works for pruning heuristics by ensuring that the *f*-value of a state is up to date before expanding it, and otherwise *reorders* it in the priority queue. Even though match pruning violates the admissibility of our heuristics for some vertices, we prove that A* is still guaranteed to find a shortest path (Supplementary Section B.2). To this end, we show that our pruning heuristics are *weakly admissible heuristics* (Supplementary Definition S7) in the sense that they are admissible on at least one path from *v_s_* to *v_t_*.

Theorem 2 A* with a weakly admissible heuristic finds a shortest path.

Theorem 3 *The pruning heuristics* ${\hat{h}}_{s}$, ${\hat{h}}_{\text{cs}}$*, and* ${\hat{h}}_{\text{gcs}}$*are weakly admissible.*

Pruning will allow us to scale near-linearly with sequence length, without sacrificing optimality of the resulting alignment.

### 3.3 Computing the heuristic

We present an algorithm to efficiently compute our heuristics (pseudocode in Supplementary Section A.4, worst-case asymptotic analysis in Supplementary Section A.5). At a high level, we rephrase the minimization of costs (over paths) to a maximization of *scores* (over chains of matches). We initialize the heuristic by precomputing all seeds, matches, potentials, and a *contours* data structure used to compute the maximum number of matches on a chain. During the A* search, the heuristic is evaluated in all explored states, and the contours are updated whenever a match gets pruned.

#### 3.3.1 Scores

The *score of a match m* is $\text{score}(m):=r-c_{m}(m)$ and is always positive. The *score of a* ${⪯}_{p}$*-chain* $m_{1}\;{⪯}_{p}\;\ldots \;{⪯}_{p}\;m_{l}$ is the sum of the scores of the matches in the chain. We define the chain score of a match *m* as

(1) $$S_{p}(m):=\underset{m{⪯}_{p}m_{1}{⪯}_{p}\ldots {⪯}_{p}m_{l}{⪯}_{p}v_{t}}{\max}\{\text{score}(m)+\cdots +\text{score}(m_{l})\}.$$

Since ${⪯}_{p}$ is a partial order, *S_p_* can be computed with base case $S_{p}(m_{\omega })=0$ and the recursion

(2) $$S_{p}(m)=\text{score}(m)+\underset{m{⪯}_{p}m\prime ⪯v_{t}}{\max}S_{p}(m\prime ).$$

We also define the chain score of a state *u* as the maximum chain score over succeeding matches *m*: $S_{p}(u)=\max_{u{⪯}_{p}m{⪯}_{p}v_{t}}S_{p}(m)$, so that Equation (2) can be rewritten as $S_{p}(m)=\text{score}(m)+S_{p}(\text{end}(m))$.

The following theorem allows us to rephrase the heuristic in terms of potentials and scores for heuristics that use $\gamma =c_{\text{seed}}$ and respect the order of the seeds, which is the case for $h_{s}$ and $h_{\text{cs}}$ (proof in Supplementary Section B.3):

Theorem 4 $h_{p,c_{\text{seed}}}^{M}(u)=P(u)-S_{p}(u)$*for any partial order* ${⪯}_{p}$*that is a refinement of* ${⪯}_{i}$*(i.e.* $u\;{⪯}_{p}\;v$*must imply* $u\;{⪯}_{i}\;v$*).*

#### 3.3.2 Layers and contours

We compute $h_{s}$ and $h_{\text{cs}}$ efficiently using *contours*. Let *layer* $L_{\ell }$ be the set of states *u* with score $S_{p}(u)\geq \ell$, so that $L_{\ell }\subseteq L_{\ell -1}$. The ℓth *contour* is the boundary of $L_{\ell }$ (Fig. 3). Layer $L_{\ell }$ (ℓ>0) contains exactly those states that precede a match *m* with score $\ell \leq S_{p}(m)<\ell +r$ (Lemma 5 in Supplementary Section B.3).

#### 3.3.3 Computing *S_p_*(*u*)

This last observation inspires our algorithm for computing chain scores. For each layer $L_{\ell }$, we store the set L[i] of matches having score ℓ: $L[\ell ]=\{m\in M\;|\;S_{p}(m)=\ell \}$. The score $S_{p}(u)$ is then the highest ℓ such that layer L[ℓ] contains a match *m* reachable from *u* ($u\;{⪯}_{p}\;m$). From Lemma 5, we know that $S_{p}(u)\geq \ell$ if and only if one of the layers L[ℓ′] for ℓ′∈[ℓ,ℓ+r) contains a match preceded by *u*. We use this to compute $S_{p}(u)$ using a binary search over the layers ℓ. We initialize $L[0]=\{m_{\omega }\}$ ($m_{\omega }$ is a fictive match at the target *v_t_*), sort all matches in M by ${⪯}_{p}$, and process them in decreasing order (from the target to the start). After computing $S_{p}(\text{end}(m))$, we add *m* to layer $S_{p}(m)=\text{score}(m)+S_{p}(\text{end}(m))$. Matches that do not precede the target (start(m) ⪯p mω) are ignored.

#### 3.3.4 Pruning matches from *L*

When pruning matches starting or ending in state *u* in layer $\ell_{u}=S_{p}(u)$, we remove all matches that start at *u* from layers $L[\ell_{u}-r+1]$ to $L[\ell_{u}]$, and all matches starting in some *v* and ending in *u* from layers $L[\ell_{v}-r+1]$ to $L[\ell_{v}]$.

Pruning a match may change *S_p_* in layers above $\ell_{u}$, so we update them after each prune. We iterate over increasing ℓ starting at $\ell_{u}+1$ and recompute $\ell \prime :=S_{p}(m)\leq \ell$ for all matches *m* in L[ℓ]. If ℓ′≠ℓ, we move *m* from L[ℓ] to L[ℓ′]. We stop iterating when either *r* consecutive layers were left unchanged, or when all matches in r−1+ℓ−ℓ′ consecutive layers have shifted down by the same amount ℓ−ℓ′. In the former case, no further scores can change, and in the latter case, *S_p_* decreases by ℓ−ℓ′ for all matches with score ≥ℓ. We remove the emptied layers L[ℓ′+1] to L[ℓ] so that all higher layers shift down by ℓ−ℓ′.

#### 3.3.5 Seed heuristic

Due to the simple structure of the SH, we also simplify its computation by only storing the start of each layer and the number of matches in each layer, as opposed to the full set of matches.

#### 3.3.6 Gap-chaining seed heuristic

Lemma 3.3 does not apply to GCSH since it uses chaining cost $\gamma =\max(c_{\text{gap}}(u,v),c_{\text{seed}}(u,v))$, which is different from $c_{\text{seed}}(u,v)$. It turns out that in this new setting it is never optimal to chain two matches if the gap cost between them is higher than the seed cost. Intuitively, it is better to miss a match than to incur additional gap-cost to include it. We capture this constraint by introducing a transformation *T* such that $u\;{⪯}_{T}\;v$ holds if and only if $c_{\text{seed}}(u,v)\geq c_{\text{gap}}(u,v)$, as shown in Supplementary Section B.4. Using an additional *consistency* constraint on the set of matches, we can compute $h_{\text{gcs}}^{M}$ via *S_T_* as before.

Definition 3 (Consistent matches) A set of matches M is *consistent* when for each m∈M (from 〈i,j〉 to 〈i′,j′〉) with score(m)>1, for each *adjacent* pair of existing states (〈i,j±1〉,〈i′,j′〉) and (〈i,j〉,〈i′,j′±1〉), there is an *adjacent* match with corresponding start and end, and score at least score(m)−1.

This condition means that for r=2, each exact match must be adjacent to four (or less around the edges of the graph) inexact matches starting or ending in the same state. Since we find all matches *m* with $c_{m}(m)<r$, our initial set of matches is consistent. To preserve consistency, we do not prune matches if that would break the consistency of M.

Definition 4 (Gap transformation) The partial order ${⪯}_{T}$ on states is induced by comparing both coordinates after the *gap transformation*

T: 〈i,j〉 ↦ (i−j−P〈i,j〉,j−i−P〈i,j〉).

Theorem 5 *Given a consistent set of matches* M*, the GCSH can be computed using scores in the transformed domain:*

hgcsM(u)={P(u)−ST(u)if u ⪯T vt,cgap(u,vt)if u ⪯T vt.

Using the transformation of the match coordinates, we reduce $c_{\text{gs}}$ to $c_{\text{seed}}$ and efficiently compute GCSH for any explored state.

## 4 Evaluations

Our algorithm is implemented in the aligner A*PA (github.com/RagnarGrootKoerkamp/astar-pairwise-aligner, tag evals) in Rust. We compare it with state-of-the-art exact aligners on synthetic (Section 4.2) and human (Section 4.3) data (github.com/pairwise-alignment/pa-bench/releases/tag/datasets) using PaBench (github.com/pairwise-alignment/pa-bench, tag astarpa-evals). We justify our heuristics and optimizations by comparing their scaling and performance (Section 4.4).

### 4.1 Setup

#### 4.1.1 Synthetic data

Our synthetic datasets are parameterized by sequence length *n*, induced error rate *e*, and total number of basepairs *N*, resulting in *N*/*n* sequence pairs. The first sequence in each pair is uniform-random from $\Sigma^{n}$. The second is generated by sequentially applying ⌊e·n⌋ edit operations (insertions, deletions, and substitutions with equal one-third probability) to the first sequence. Introduced errors can cancel each other, making the *divergence d* between the sequences less than *e*. Induced error rates of 1%, 5%, 10%, and 15% correspond to divergences of 0.9%, 4.3%, 8.2%, and 11.7%, which we refer to as 1%, 4%, 8%, and 12%.

#### 4.1.2 Human data

We use two datasets of ultra-long ONT reads of the human genome: one without and one with genetic variation. All reads are 500–1100kb long, with mean divergence around 7%. The average length of the longest gap in the alignment is 0.1kb for ONT reads, and 2kb for ONT reads with genetic variation (detailed statistics in Supplementary Section C.5). The reference genome is CHM13 (v1.1) (Nurk *et al.* 2022). The reads used for each dataset are:

- *ONT*: 50 reads sampled from those used to assemble CHM13.
- *ONT with genetic variation*: 48 reads from another human (Bowden *et al.* 2019), as used in the BiWFA paper (Marco-Sola *et al.* 2023).

#### 4.1.3 Algorithms and aligners

We compare SH, CSH, and GCSH (all with pruning) as implemented in A*PA to the state-of-the-art exact aligners BiWFA and Edlib. We also compare to Dijkstra’s algorithm and A* with previously introduced heuristics (gap cost and character frequencies of Hadlock (1988a), and SH without pruning of Ivanov *et al.* (2022)). We exclude SeqAn and Parasail since they are outperformed by WFA and Edlib (Šošić and Šikić 2017, Marco-Sola *et al.* 2021). We run all aligners with unit edit costs with traceback enabled.

#### 4.1.4 A*PA parameters

Inexact matches (r=2) and short seeds (low *k*) increase the accuracy of GCSH for divergent sequences, thus reducing the number of expanded states. On the other hand, shorter seeds have more matches, slowing down precomputation and contour updates. A parameter grid search on synthetic data (Supplementary Section C.1) shows that the runtime is generally insensitive to *k* as long as *k* is high enough to avoid too many spurious matches ($k\gg {\;\text{log}}_{4}\;n$), and the potential is sufficiently larger than edit distance (k≪r/d). For d=4%, exact matches lead to faster runtimes, while d=12% requires r=2 and k<2/d=16.7. We fix *k *=* *15 throughout the evaluations since this is a good choice for both synthetic and human data.

#### 4.1.5 Execution

We use PaBench on Arch Linux on an Intel Core i7-10750H processor with 64 GB of memory and 6 cores, without hyper-threading, frequency boost, and CPU power saving features. We fix the CPU frequency to 2.6 GHz, limit the memory usage to 32 GiB, and run one single-threaded job at a time with niceness –20.

#### 4.1.6 Measurements

Pa Bench first reads the dataset from disk and then measures the wall-clock time and increase in memory usage of each aligner. Plots and tables refer to the average alignment time per aligned pair, and for A*PA include the time to build the heuristic. Best-fit polynomials are calculated via a linear fit in the log–log domain using the least squares method.

### 4.2 Scaling on synthetic data

#### 4.2.1 Runtime scaling with length

**Figure 4.**
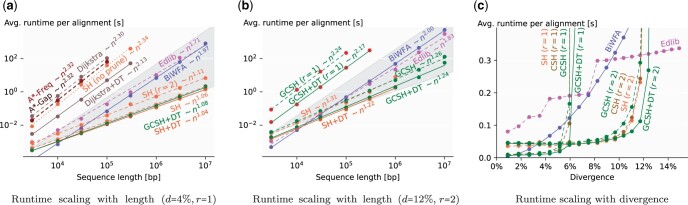
Runtime comparison on synthetic data. (a, b) Log–log plots comparing variants of our heuristic, including the simplest (SH) and most accurate (GCSH with DT), to Edlib, BiWFA, and other algorithms (averaged over 10^6^–10^7^ total bp, seed length k=15). The slopes of the bottom (top) of the background cones correspond to linear (quadratic) growth. SH without pruning is dotted, and variants with DT are solid. For d=12%, red dots show where the heuristic potential is less than the edit distance. Missing data points are due to exceeding the 32 GB memory limit. (c) Runtime scaling with divergence ($n=10^{5}$, 10^6^ total bp, and k=15).

We compare our A* heuristics with Edlib, BiWFA, and other heuristics in terms of runtime scaling with *n* and *d* (Fig. 4, extended comparison in Supplementary Section C.2). As theoretically predicted, Edlib and BiWFA scale quadratically. For small edit distance, Edlib is subquadratic due to the bit-parallel optimization. Dijkstra, A* with the gap heuristic, character frequency heuristic (Hadlock 1988a), or original SH (Ivanov *et al.* 2022) all scale quadratically. The empirical scaling of A*PA is subquadratic for d≤12 and $n\leq 10^{7}$, making it the fastest aligner for long sequences (n>30kb). For low divergence (d≤4%) even the simplest SH scales near-linearly with length (best fit $n^{1.06}$ for $n\leq 10^{7}$). For high divergence (d=12%), we need inexact matches, and the runtime of SH sharply degrades for long sequences ($n>10^{6}\;\text{bp}$) due to spurious matches. This is countered by chaining the matches in CSH and GCSH, which expand linearly many states (Supplementary Section C.3). GCSH with DT is not exactly linear due to high memory usage and state reordering (Supplementary Section C.7 shows the time spent on parts of the algorithm).

#### 4.2.2 Runtime scaling with divergence

Figure 3c shows that A*PA has near constant runtime in *d* as long as the edit distance is sufficiently less than the heuristic potential (i.e. d≪r/k). In this regime, A*PA is faster than both Edlib (linear in *d*) and BiWFA (quadratic in *d*). For 1≤d≤6%, exact matches have less overhead than inexact matches, while BiWFA is fastest for d≤1%. A*PA becomes linear in *d* for d≥r/k (Supplementary Section C.4).

#### 4.2.3 Performance

A*PA with SH with DT is >500× faster than Edlib and BiWFA for d=4% and $n=10^{7}$ (Fig. 4a). For $n=10^{6}$ and d≤12%, memory usage is <500 MB for all heuristics (Supplementary Section C.6).

### 4.3 Speedup on human data

We compare runtime (Fig. 4 and Supplementary Section C.7), and memory usage (Supplementary Section C.6) on human data. We configure A*PA to prune matches only when expanding their start (not their end), leaving some matches on the optimal path unpruned and speeding up contour updates. The runtime of A*PA (GCSH with DT) on ONT reads is less than Edlib and BiWFA in all quartiles, with the median being >3× faster. However, the runtime of A*PA grows rapidly when d≥10%, so we set a time limit of 100 s per read, causing six alignments to time out. In real-world applications, the user would either only get results for a subset of alignments, or could use a different tool to align divergent sequences. With genetic variation, A*PA is 1.7× faster than Edlib and BiWFA in median. Low-divergence alignments are faster than Edlib, while high-divergence alignments are slower (three sequences with d≥10% time out) because of expanding quadratically many states in complex regions (Supplementary Section C.8). Since slow alignments dominate the total runtime, Edlib has a lower mean runtime.

### 4.4 Effect of pruning, inexact matches, chaining, and DT

We visualize our techniques on a complex alignment in Supplementary Section C.10.

#### 4.4.1 SH with pruning enables near-linear runtime

Figure 3a shows that the addition of match pruning changes the quadratic runtime of SH without pruning to near-linear, giving multiple orders of magnitude speedup.

#### 4.4.2 Inexact matches cope with higher divergence

Inexact matches double the heuristic potential, thereby almost doubling the divergence up to which A*PA is fast (Fig. 4c). This comes at the cost of a slower precomputation to find all matches.

#### 4.4.3 Chaining copes with spurious matches

While CSH improves on SH for some very slow alignments (Fig. 4), more often the overhead of computing contours makes it slower than SH.

#### 4.4.4 Gap-chaining copes with indels

**Figure 5.**
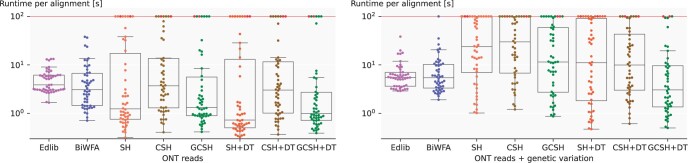
Runtime on long human reads. Each dot is an alignment without (left) or with (right) genetic variation. Runtime is capped at 100 s. Boxplots show the three quartiles and red dots show where the edit distance is larger than the heuristic potential. The median runtime of A*PA (GCSH + DT, k=15, r=2) is 3× (left) and 1.7× (right) faster than Edlib and BiWFA.

GCSH is significantly and consistently faster than SH and CSH on human data, especially for slow alignments (Fig. 5). GCSH has less overhead over SH than CSH, due to filtering out matches m⪯vt.

#### 4.4.5 DT speeds up quadratic search

DT significantly reduces the number of expanded states when the A* search is quadratic (Fig. 4a and Supplementary Section C.4). This results in a significant speedup for genetic variation of long indels (Fig. 5).

CSH, GCSH, and DT only have a small impact on the uniform synthetic data, where usually either the SH is sufficiently accurate for the entire alignment and runtime is near-linear (d≪r/k), or even GCSH is not strong enough and runtime is quadratic (d≫r/k). On human data however, containing longer indels and small regions of quadratic search, the additional accuracy of GCSH and the reduced number of states explored by DT provide a significant speedup (Supplementary Section C.10).

## 5 Discussion

### 5.1 Seeds are necessary; matches are optional

The SH exploits the lack of matches to penalize alignments. In our heuristics, the more seeds without matches, the higher the penalty for alignments and the easier it is to dismiss suboptimal ones. In the extreme, not having any matches can be sufficient for finding an optimal alignment in linear time (Supplementary Section C.9).

### 5.2 Modes: near-linear and quadratic

The A* algorithm with a SH has two modes of operation that we call *near-linear* and *quadratic*. In the near-linear mode, A*PA expands few vertices because the heuristic successfully penalizes all edits between the sequences. When the divergence is larger than what the heuristic can handle, every edit that is not penalized by the heuristic increases the explored band, leading to a quadratic exploration similar to Dijkstra.

### 5.3 Limitations

1. *Quadratic scaling.* Complex data can trigger a quadratic (Dijkstra-like) search, which nullifies the benefits of A* (Supplementary Sections C.8 and C.10). Regions with high divergence (d≥10%), such as high error rate or long indels, exceed the heuristic potential to direct the search and make the exploration quadratic. Low-complexity regions (e.g. with repeats) result in a quadratic number of matches, which also take quadratic time.
2. Computational overhead of A*. Computing states sequentially (as in Edlib, BiWFA) is orders of magnitude faster than computing them in random order (as in Dijkstra, A*). A*PA outperforms Edlib and BiWFA (Fig. 4a) when the sequences are long enough for the linear-scaling to have an effect (n>30kb), and there are enough errors (d>1%) to trigger the quadratic behaviour of BiWFA.

### 5.4 Future work

1. *Performance.* We are working on a DP version of A*PA that applies computational volumes (Spouge 1989, 1991), block-based computations (Liu and Steinegger 2023), and a SIMD version of Edlib’s bit-parallelization (Myers 1999). This has already shown 10× additional speedup on the human datasets and is less sensitive to the input parameters. Independently, the number of matches could be reduced by using variable seed lengths and skipping seeds having many matches.
2. *Generalizations.* Our CSH could be generalized to non-unit and affine costs, and to semi-global alignment. Cost models that better correspond to the data can speed up the alignment.
3. *Relaxations.* At the expense of optimality guarantees, inadmissible heuristics could speed up A*. Another possible relaxation would be to validate the optimality of a given alignment instead of computing it from scratch.
4. *Analysis.* The near-linear scaling with length of A* is not asymptotic and requires a more thorough theoretical analysis (Medvedev 2023b).

## Supplementary Material

btae032_Supplementary_Data

## Data Availability

The data underlying this article are available on Github at https://github.com/pairwise-alignment/pa-bench/releases/tag/datasets.
