# Supplementary material for: Exact global alignment using A* with chaining seed heuristic and match pruning
Source: Bioinformatics. 2024 Jan 23;40(3):btae032. doi: 10.1093/bioinformatics/btae032 (PMC10932610; doi:10.1093/bioinformatics/btae032)
Supplement: btae032_Supplementary_Data [file btae032_supplementary_data.pdf]

A. Pseudocode

A.1. A* algorithm for match pruning

We present our variant of A* (Hart et al., 1968) that supports match pruning (Algorithm 1). All computed values of g are stored in a hash map, and all *open* states are stored in a bucket queue of tuples $(v, g(v), f(v))$ ordered by increasing f. Line 14 prunes (removes) a match and thereby increases some heuristic values before that match. As a result, some f-values in the priority queue may become outdated. Line 11 solves this problem by checking if the f-value of the state about to be expanded was changed, and if so, line 12 pushes the updated state to the queue, and proceeds by choosing a next best state. This way, we guarantee that the expanded state has minimal updated f. To reconstruct the best alignment we traceback from the target state using the hash map g (not shown).

A.2. Diagonal transition for A*

For a given distance g, the diagonal transition method only considers the *farthest-reaching* (f.r.) state $u{=}\langle i,j\rangle$ on each diagonal $k{=}i{-}j$ at distance g. We use $F_{gk} := i{+}j$ to indicate the antidiagonal[4] of the farthest reaching state. Let X_{gk} be the farthest state on diagonal k adjacent to a state at distance $g{-}1$, which is then *extended* into F_{gk} by following as many matches as possible. The edit distance is the lowest g such that $F_{g,n-m} \geq n+m$, and we have the recursion

$$X_{gk} := \max(F_{g-1,k-1}{+}1, F_{g-1,k}{+}2, F_{g-1,k+1}{+}1), \tag{3}$$

$$F_{gk} = X_{gk} + \mathrm{LCP}\left(A_{\geq(X_{gk}+k)/2}, B_{\geq(X_{gk}-k)/2}\right). \tag{4}$$

[4] Previous works indicate the column i of u, but using the antidiagonal $i{+}j$ keeps the symmetry between insertions and deletions.

Algorithm 1 A* algorithm with match pruning.
Lines added for pruning (11, 12, and 14) are marked in **bold**.

1: **Input:** Sequences A and B and pruning heuristic h
2: **Output:** Edit distance between A and B
3: **function** ASTAR(A, B, h)
4: $g(v_s) \leftarrow 0$ ▷ Hashmap of distances; default $+\infty$
5: $q \leftarrow BucketQueue()$ ▷ Bucket queue of open states
6: $q.push((v_s, g{=}0, f{=}0))$
7: **repeat**
8: $(u, g_u, f_u) \leftarrow q.pop()$ ▷ Pop u with minimal f
9: **if** $g_u > g(u)$ **then**
10: **continue** ▷ u was already expanded
11: **else if** $f_u < g(u) + h(u)$ **then** ▷ $h(u)$ has increased
12: $q.push((u, g_u, g(u) + h(u)))$ ▷ *Reorder* u
13: **else** ▷ *Expand* u
14: $Prune(u)$
15: **for** successors v of u **do**
16: $g_v \leftarrow g_u + \mathrm{d}(u,v)$
17: $v \leftarrow Extend(v)$ ▷ Greedy matching within seed
18: **if** $g_v < g(v)$ **then** ▷ *Open* v
19: $g(v) \leftarrow g_v$
20: $q.push((v, g_v, g_v + h(v)))$
21: **until** v_t is expanded
22: **return** $g(v_t)$

The base case is $X_{0,0}{=}0$ with default value $F_{gk}{=}{-}\infty$ for $k{>}|g|$, and LCP is the length of the longest common prefix of two strings. Each edge in a traceback path is either a match created by an extension (4), or a mismatch starting in a f.r. state (3). We call such a path an *f.r. path*.

Implementation. In Algorithm 2 we further modify the A* algorithm to only consider f.r. paths. We replace the map g that tracks the best distance by a map F_{gk} that tracks f.r. states (lines 4, 18, and 19). Instead of $g(u)$ decreasing over time, we now ensure that $F_{g,k}$ increases over time. Each time a state u is opened or expanded, the check whether $g(u)$ decreases is replaced by a check whether F_{gk} increases (line 9). This causes the search to skip states that are known to not be farthest reaching. The proof of correctness (Thm. 2) still applies.

Alternatively, it is also possible to implement A* directly in the diagonal-transition state-space by pushing states F_{gk} to the priority queue, but for simplicity we keep the similarity with the original A*.

A.3. Implementation notes

Here we list implementation details on performance and correctness.

Bucket queue. We use a hashmap to store all computed values of g in the A* algorithm. Since the edit costs are bounded integers, we implement the priority queue using a *bucket queue* (Hitchner, 1969; Dial, 1969; Bertsekas, 1991). Unlike heaps, this data structure has amortized constant time push and pop operations since the value difference between consecutive pop operations is bounded.

Greedy matching of letters. From a state $\langle i,j\rangle$ where $a_i = b_j$, it is sufficient to only consider the matching edge to $\langle i{+}1,j{+}1\rangle$ (Allison, 1992; Ivanov et al., 2020), and ignore

Algorithm 2 A*-DT algorithm with match pruning.
Lines changed for diagonal transition (4, 9, 18, and 19) are in **bold**.

1: **Input:** Sequences A, B and pruning heuristic h
2: **Output:** Edit distance between A and B
3: **function** ASTAR-DT(A, B, h)
4: $F_{0,0} \leftarrow 0$ ▷ Hashmap of f.r. point per g and k; default $-\infty$
5: $q \leftarrow BucketQueue()$ ▷ Bucket queue of open states
6: $q.push((v_s, g{=}0, f{=}0))$
7: **repeat**
8: $(u, g_u, f_u) \leftarrow q.pop()$ ▷ Pop u with minimal f
9: **if** $i_u{+}j_u < F_{g_u, i_u-j_u}$ **then**
10: **continue** ▷ u is not farthest reaching
11: **else if** $f_u < g(u) + h(u)$ **then** ▷ $h(u)$ has increased
12: $q.push((u, g_u, g(u) + h(u)))$ ▷ *Reorder* u
13: **else** ▷ *Expand* u
14: $Prune(u)$
15: **for** successors v of u **do**
16: $g_v \leftarrow g_u + \mathrm{d}(u,v)$
17: $v \leftarrow Extend(v)$ ▷ Greedy matching in seed
18: **if** $i_v{+}j_v > F_{g_v, i_v-j_v}$ **then** ▷ *Open* v
19: $F_{g_v, i_v-j_v} \leftarrow i_v{+}j_v$
20: $q.push((v, g_v, g_v + h(v)))$
21: **until** v_t is expanded
22: **return** $g(v_t)$

the insertion and deletion edges to $\langle i, j{+}1\rangle$ and $\langle i{+}1, j\rangle$. During alignment, we greedily match as many letters as possible within the current seed before inserting only the last open state in the priority queue, but we do not cross seed boundaries in order to not interfere with match pruning. This optimization is superseded by the DT-optimization. We include greedily matched states in the reported number of expanded states.

Priority queue offset. Pruning the last remaining match in a layer may cause an increase of the heuristic in all states preceding the start u of the match. This invalidates f values in the priority queue and causes reordering. We skip most of the update operations by storing a global offset to the f-values in the priority queue, which we update when all states in the priority queue precede u.

Split vector for layers. Pruning a match may trigger the removal of one or more layers of matches in L, and the shifting down of higher layers. To efficiently remove layers, we use a *split vector* data structure consisting of two stacks. In the first stack we store the layers before the last deleted layer, and in the second stack the remaining layers in reverse order. Before deleting a layer, we move layers from the top of one of the stacks to the top of the other, until the layer to be deleted is at the top of one of the stacks. Removing layers in decreasing order of ℓ takes linear total time.

Binary search speedup. Instead of using binary search to determine the layer/score $S_p(u)$ (Algorithm 3), we first try a linear search around either the score of the parent of u or a known previous score at u. This linear search usually finds the correct layer in a few iterations, or otherwise we fall back to binary search.

In practice, most pruning happens near the tip of the search, and the number of layers between the start v_s and an open state u rarely changes. Thus, to account for changing scores, we store a *hint* of value $S_p(v_s) - S_p(u)$ in a hashmap and start the search at $S_p(v_s) - hint$.

Code correctness. Our implementation A*PA is written in Rust, contains many assertions testing e.g. the correctness of our A* and layers data structure implementation, and is tested for correctness and performance on randomly-generated sequences. Correctness is checked against simpler algorithms (Needleman-Wunsch) and other aligners (EDLIB, BIWFA).

A.4. Computation of the heuristic

Algorithm 3 shows how the heuristic is initialized, how $S_p(u)$ and $h(u)$ are computed, and how matches are pruned.

Algorithm 3 Computation of the heuristic.

Input: Sequences A and B
Output: Heuristic h
function INITIALIZEHEURISTIC(A, B, k)
  $S \leftarrow$ Non-overlapping k-mers of A ▷ Seeds
  $H \leftarrow$ Map of all k-mers (and k±1-mers for $r = 2$) of B
  $\mathcal{M}_s \leftarrow \bigcup_{s':\ \mathrm{ed}(s,s')<r} H[s']$ ▷ Seed matches
  $\mathcal{M} \leftarrow \bigcup_{s\in\mathcal{S}} \mathcal{M}_s$ ▷ Matches
  $P(i) \leftarrow r \cdot |S_{\geq i}|$ for all $i \in [0, |A|]$ ▷ Potentials
  $L[0] = \{m_\omega\}$ ▷ Layers
  for $m \in \mathcal{M}$ in decreasing order of $\preceq_p$ **do**
    $\ell \leftarrow \mathrm{score}(m) + S_p(\mathrm{end}(m))$
    if $m \preceq_p m_\omega$ **then** ▷ If m precedes the target
      $L[\ell].push(m)$

function $S_p(u)$
  function TEST(ℓ)
    for $\ell' \in [\ell, \ell + r)$ **do**
      if $u \preceq_p m$ for some $m \in L[\ell']$ **then**
        return $True$
    return $False$
  return $\max\{\ell \mid \mathtt{TEST}(\ell)\}$ ▷ Binary search the highest ℓ

function $h_{\mathrm{s}}(u)$ ▷ SH
  return $P(u) - S_i(u)$

function $h_{\mathrm{cs}}(u)$ ▷ CSH
  return $P(u) - S_{\preceq}(u)$

function $h_{\mathrm{gcs}}(u)$ ▷ GCSH
  return $\max(P(u) - S_T(u), c_{\mathrm{gap}}(u, v_t))$

function PRUNE(u)
  $\ell_u \leftarrow S_p(u)$
  for all $m \in \mathcal{M}$: $start(m) = u$ **or** $end(m) = u$ **do**
    if $h \neq h_{\mathrm{gcs}}$ **or** $\mathcal{M}\backslash\{m\}$ is consistent **then**
      Remove m from $\mathcal{M}$
      for $\ell \in [S_p(start(m)) - r + 1, S_p(start(m))]$ **do**
        Remove m from $L[\ell]$ if present
  for $\ell \in [\ell_u + 1, S_p\langle 0, 0\rangle]$ **do**
    for all $m \in L[\ell]$ **do**
      $\ell' \leftarrow S_p(m)$
      if $\ell' \neq \ell$ **then**
        Move m from $L[\ell]$ to $L[\ell']$
    if r consecutive layers unchanged **then**
      return
    if $r{-}1 + \ell - \ell'$ consecutive layers shifted exactly $\ell{-}\ell'$ **then**
      Remove empty layers $L[\ell'{+}1], \ldots, L[\ell]$ ▷ Shift higher layers down
      return

A.5. Worst-case runtime asymptotics

Our algorithms optimize for the average case performance. Nevertheless, we discuss the worst-case time of each part of the algorithm. To analyze the worst-case asymptotics of A*PA, let $M = O(n^2)$ be the number of (inexact) matches, and let $E = O(n^2)$ be the number of expanded states. Then the asymptotical runtime of our algorithm breaks down as:

1. Finding all matches takes $O(n + M)$ time.
2. Building the contours datastructure takes $O(M \log M)$ time.
3. Each expanded state is pushed onto and popped from the priority queue ($O(1)$ using a bucket queue), looked up in a hashmap ($O(1)$), and has its neighbours explored ($O(1)$), for $O(E)$ total.
4. Evaluating the heuristic requires a binary search over contours, which is bounded by $O(n \lg n)$, since we test at most $\lg n$ contours each containing at most n matches. (This could be improved to $O(\log^2 n)$ by storing each contour as an ordered set, but it is not usually a bottleneck.)
5. Pruning a match requires a dictionary lookup and change for $O(1)$. Updating contours after pruning requires iterating over higher contours until no more changes are triggered. This could

take $O(M)$ for each match that is pruned, leading to a naive upper bound of $O(M^2)$.

6. Reordering states takes $O(En)$, since the heuristic in each expanded state can decrease at most n times.
7. The traceback requires $O(n)$ time.

Taking this together, we obtain $O(n + M \log M + En \lg n + M^2 + En)$. When the number of matches M is $\Theta(n^2)$, the updating of contours has the worst upper bound and the asymptotics becomes $O(n^4)$. Whether this upper bound can be reached in practice or whether a tigher bound exists needs further investigation. Even if the worst-case was $\Theta(n^2)$, in practice our algorithm only runs efficiently in regimes well below this bound.

Sec. C.1 discusses the effect of r and k on the number of expanded states when aligning synthetic data, and Sec. C.4 discusses the effect of the divergence d on the number of expanded states.

B. Proofs

B.1. Admissibility

Our heuristics are not *consistent*, but we show that a weaker variant holds for states *at the start of a seed.*

Definition 5 (Start of seed) A state $\langle i, j\rangle$ is *at the start of some seed* when i is a multiple of the seed length k, or when $i = n$.

Lemma 1 (Weak triangle inequality) *For states u, v, and w with v and w at the starts of some seeds, all $\gamma \in \{c_{\text{seed}}, c_{\text{gap}}, c_{\text{gs}}\}$ satisfy*

$$\gamma(u, v) + \gamma(v, w) \geq \gamma(u, w).$$

Proof Both v and w are at the start of some seeds, so for $\gamma = c_{\text{seed}}$ we have the equality $c_{\text{seed}}(u, w) = c_{\text{seed}}(u, v) + c_{\text{seed}}(v, w)$.

For $\gamma = c_{\text{gap}}$,

$$\begin{aligned} & c_{\text{gap}}(\langle i, j\rangle, \langle i', j'\rangle) + c_{\text{gap}}(\langle i', j'\rangle, \langle i'', j''\rangle) \\ &= |(i' - i) - (j' - j)| + |(i'' - i') - (j'' - j')| \\ &\geq |(i'' - i) - (j'' - j)| = c_{\text{gap}}(\langle i, j\rangle, \langle i'', j''\rangle). \end{aligned}$$

And lastly, for $\gamma = c_{\text{gs}}$,

$$\begin{aligned} & c_{\text{gs}}(u, v) + c_{\text{gs}}(v, w) \\ &= \max(c_{\text{gap}}(u, v), c_{\text{seed}}(u, v)) + \max(c_{\text{gap}}(v, w), c_{\text{seed}}(v, w)) \\ &\geq \max(c_{\text{gap}}(u, v) + c_{\text{gap}}(v, w), c_{\text{seed}}(u, v) + c_{\text{seed}}(v, w)) \\ &\geq \max(c_{\text{gap}}(u, w), c_{\text{seed}}(u, w)) = c_{\text{gs}}(u, w). \end{aligned}$$ □

Lemma 2 (Weak consistency) *Let $h \in \{h^{\mathcal{M}}_{\text{s}}, h^{\mathcal{M}}_{\text{cs}}, h^{\mathcal{M}}_{\text{gcs}}\}$ be a heuristic with partial order $\preceq_p$, and let $u \preceq_p v$ be states with v at the start of a seed. When there is a shortest path π^* from u to v such that $\mathcal{M}$ contains all matches of cost less than r on π^*, it holds that $h(u) \leq \mathrm{d}(u, v) + h(v)$.*

Proof The path π^* covers each seed in $\mathcal{S}_{u\dots v}$ that must to be fully aligned between u and v. Since the seeds do not overlap, their shortest alignments π^*_s in π^* do not have overlapping edges. Let $u \leq m_1 \preceq_p \cdots \preceq_p m_l \preceq_p v$ be the chain of matches $m_i \in \mathcal{M}$ corresponding to those π^*_s of cost less than r (Fig. 6). Since the matches and the paths between them are disjoint, $\mathrm{c}_{\text{path}}(\pi^*)$ is at least the cost of the matches $\mathrm{c}_{\text{m}}(m_{i+1}) = \mathrm{d}(\text{start}(m_{i+1}), \text{end}(m_{i+1}))$ plus the cost to chain these matches $\gamma(\text{end}(m_i), \text{start}(m_{i+1})) \leq \mathrm{d}(\text{end}(m_i), \text{start}(m_{i+1}))$. Putting this together:

$$\begin{aligned} & \gamma(u, m_1) + \mathrm{c}_{\text{m}}(m_1) + \cdots + \mathrm{c}_{\text{m}}(m_l) + \gamma(m_l, v) \\ \leq\ & \mathrm{d}(u, \text{start}(m_1)) + \mathrm{d}(\text{start}(m_1), \text{end}(m_1)) + \cdots + \mathrm{d}(\text{end}(m_l), v) \\ \leq\ & \mathrm{d}(u, v). \end{aligned}$$

Now let $v \preceq_p m_{l+1} \preceq_p \cdots \preceq_p m_{l'} \preceq_p v_t$ be a chain of matches minimizing $h(v)$ (Def. 1) with $w := \text{start}(m_{l+1})$. This chain also minimizes $h(w)$ and thus $h(v) = \gamma(v, w) + h(w)$. We can now bound the cost of the joined chain from u to v and from w to the end and get our result via $\gamma(m_l, w) \leq \gamma(m_l, v) + \gamma(v, w)$ (Lemma 1)

$$\begin{aligned} h(u) &\leq \gamma(u, m_1) + \cdots + \gamma(m_l, m_{l+1}) + \mathrm{c}_{\text{m}}(m_{l+1}) + \cdots + \gamma(m_{l'}, v_t) \\ &= \gamma(u, m_1) + \cdots + \gamma(m_l, w) \qquad + h(w) \\ &\leq \gamma(u, m_1) + \cdots + \gamma(m_l, v) + \gamma(v, w) + (h(v) - \gamma(v, w)) \\ &\leq \mathrm{d}(u, v) + h(v). \end{aligned}$$ □

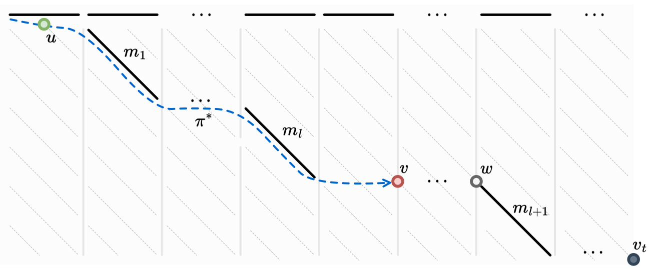

Fig. 6: **Variables of the proof of Lemma 2.**

Theorem 1 *The seed heuristic h_{s}, the chaining seed heuristic h_{cs}, and the gap-chaining seed heuristic h_{gcs} are* admissible. *Furthermore, $h_{\mathrm{s}}^{\mathcal{M}}(u) \le h_{\mathrm{cs}}^{\mathcal{M}}(u) \le h_{\mathrm{gcs}}^{\mathcal{M}}(u)$ for all states u.*

Proof We will prove $h_{\mathrm{s}}^{\mathcal{M}}(u) \overset{(1)}{\le} h_{\mathrm{cs}}^{\mathcal{M}}(u) \overset{(2)}{\le} h_{\mathrm{gcs}}^{\mathcal{M}}(u) \overset{(3)}{\le} h^*(u)$, which implies the admissibility of all three heuristics.

(1) Note that $u \le v$ implies $u \le_i v$ and hence any $\le$-chain is also a $\le_i$-chain. A minimum over the superset of $\le_i$-chains is at most the minimum of the subset of $\le$-chains, and hence $h_{\mathrm{s}}^{\mathcal{M}} = h_{\le_i, c_{\mathrm{seed}}}^{\mathcal{M}} \le h_{\le, c_{\mathrm{seed}}}^{\mathcal{M}} = h_{\mathrm{cs}}^{\mathcal{M}}$.

(2) The only difference between $h_{\mathrm{cs}}^{\mathcal{M}}$ and $h_{\mathrm{gcs}}^{\mathcal{M}}$ is that the former uses c_{seed} and the latter uses the gap-seed cost $c_{\mathrm{gs}} := \max(c_{\mathrm{gap}}, c_{\mathrm{seed}})$. Since $c_{\mathrm{seed}} \le c_{\mathrm{gs}}$ we have $h_{\mathrm{cs}}^{\mathcal{M}} = h_{\le, c_{\mathrm{seed}}}^{\mathcal{M}} \le h_{\le, c_{\mathrm{gs}}}^{\mathcal{M}} = h_{\mathrm{gcs}}^{\mathcal{M}}$.

(3) When $\mathcal{M}$ is the set of all matches with costs strictly less than r, admissibility follows directly from Lemma 2 with $v = v_t$ via

$$h_{\mathrm{gcs}}^{\mathcal{M}}(u) \le \mathrm{d}(u, v_t) + h_{\mathrm{gcs}}^{\mathcal{M}}(v_t) = \mathrm{d}(u, v_t) = h^*(u). \qquad \square$$

B.2. Match pruning

During the A* search, we continuously improve our heuristic using match pruning. The pruning increases the value of our heuristics and breaks their admissibility. Nevertheless, we prove in two steps that A* with match pruning still finds a shortest path. First, we introduce the concept of a *weakly-admissible heuristic* and show that A* using a weakly-admissible heuristic finds a shortest path (Thm. 2). Then, we show that our pruning heuristics are indeed weakly admissible (Thm. 3).

A* with a weakly-admissible heuristic finds a shortest path.

Definition 6 (Fixed vertex) A vertex u is *fixed* if it is expanded and A* has found a shortest path to it, that is, $g(u) = g^*(u)$.

A fixed vertex cannot be opened again (Algorithm 1, line 18), and hence remains fixed.

Definition 7 (Weakly admissible) A heuristic $\hat{h}$ is *weakly admissible* if at any moment during the A* search there exists a shortest path π^* from v_s to v_t in which all vertices $u \in \pi^*$ after its last fixed vertex n^* satisfy $\hat{h}(u) \le h^*(u)$.

To prove that A* finds a shortest path when used with a weakly-admissible heuristic, we follow the structure of Hart et al. (1968). First we restate their Lemma 1 in our notation with a slightly stronger conclusion that follows directly from their proof.

Lemma 3 (Lemma 1 of Hart et al. (1968)) *For any unfixed vertex n and for any shortest path π^* from v_s to n, there exists an open vertex n' on π^* with $g(n') = g^*(n')$ such that π^* does not contain fixed vertices after n'.*

Next, we prove that in each step the A* algorithm can proceed along a shortest path to the target:

Corollary 1 (Generalization of Corollary to Lemma 1 of Hart et al. (1968)) *Suppose that $\hat{h}$ is weakly admissible, and that A* has not terminated. Then, there exists an open vertex n' on a shortest path from v_s to v_t with $f(n') \le g^*(v_t)$.*

Proof Let π^* be the shortest path from v_s to v_t given by the weak admissibility of $\hat{h}$ (Def. 7). Since A* has not terminated, v_t is not fixed. Substitute $n = v_t$ in Lemma 3 to derive that there exists an open vertex n' on π^* with $g(n') = g^*(n')$. By definition of f we have $f(n') = g(n') + \hat{h}(n')$. Since π^* does not contain any fixed vertices after n', the weak admissibility of $\hat{h}$ implies $\hat{h}(n') \le h^*(n')$. Thus, $f(n') = g(n') + \hat{h}(n') \le g^*(n') + h^*(n') = g^*(v_t)$. $\square$

Theorem 2 *A* with a weakly-admissible heuristic finds a shortest path.*

Proof The proof of Theorem 1 in Hart et al. (1968) applies, with the remark that instead of an arbitrary shortest path, we use the specific path π^* given by the weak admissibility and the specific vertex n' given by **??** 1. $\square$

Our heuristics are weakly admissible. A *consistent* heuristic finds the correct distance to each vertex as soon as it is expanded. While our heuristics are not consistent, this property is true for states *at the starts of seeds* (when i is a multiple of the seed length k, or when $i = n$).

Lemma 4 *For $\hat{h} \in \{\hat{h}_{\mathrm{s}}, \hat{h}_{\mathrm{cs}}, \hat{h}_{\mathrm{gcs}}\}$, every state at the start of a seed becomes fixed immediately when A* expands it.*

Proof We use a proof by contradiction: suppose that v is a state at the start of some seed that is expanded but not fixed. In other words, $f(v)$ is minimal among all open states, but the shortest path π^* from v_s to v has strictly smaller length $g^*(v) < g(v)$.

Let n^* be the last fixed state on π^* before v, and let $u \in \pi^*$ be the successor of n^*. State u is open because its predecessor n^* is fixed and on a shortest path to u. Let the chain of all matches of cost less than r on π^* between u and v be $u \le m_1 \le \cdots \le m_l \le v$. Since n^* is the last fixed state on π^*, none of these matches has been pruned, and they are all in $\mathcal{M}\backslash E$ as well. This means we can apply Lemma 2 to get $h(u) \le \mathrm{d}(u, v) + h(v)$, so

$$\begin{aligned}
f(u) = g(u) + h(u) &= g^*(u) + h(u) && \pi^* \text{ is a shortest path}\\
&\le g^*(u) + \mathrm{d}(u, v) + h(v) && \text{shown above}\\
&= g^*(v) + h(v) && \pi^* \text{ is a shortest path}\\
&< g(v) + h(v) = f(v) && \text{by assumption.}
\end{aligned}$$

This proves that $f(u) < f(v)$, resulting in a contradiction with the assumption that v is an open state with minimal f. $\square$

Theorem 3 *The pruning heuristics $\hat{h}_{\mathrm{s}}$, $\hat{h}_{\mathrm{cs}}$, $\hat{h}_{\mathrm{gcs}}$ are weakly admissible.*

Proof Let π^* be a shortest path from v_s to v_t. At any point during the A* search, let n^* be the farthest expanded state on π^* that is at the start of a seed. By Lemma 4, n^* is fixed. By the choice of n^*, no states on π^* after n^* that are at the start of a seed are expanded, so no matches on π^* following n^* are pruned. Now the proof of Thm. 1 applies to the part of π^* after n^* without changes, implying that $\hat{h}(u) \le h^*(u)$ for all u on π^* following n^*, for any $\hat{h} \in \{\hat{h}_{\mathrm{s}}, \hat{h}_{\mathrm{cs}}, \hat{h}_{\mathrm{gcs}}\}$. $\square$

B.3. Computation of the (chaining) seed heuristic

Theorem 4 $h^{\mathcal{M}}_{p,c_{\text{seed}}}(u) = P(u) - S_p(u)$ *for any partial order* $\preceq_p$ *that is a refinement of* $\preceq_i$ *(i.e.* $u \preceq_p v$ *must imply* $u \preceq_i v$*).*

Proof For a chain of matches $\{m_i\} \subseteq \mathcal{M}$, let s_i and t_i be the start and end states of m_i. We translate the terms of our heuristic from costs to potentials and match scores (Sec. 3.3):

$$\begin{aligned} & c_{\text{seed}}(m_i, m_{i+1}) \;+\; c_{\text{m}}(m_{i+1}) \\ = \;& \big(P(t_i) - P(s_{i+1})\big) \;+\; \big(P(s_{i+1}) - P(t_{i+1}) - \text{score}(m_{i+1})\big) \\ = \;& P(t_i) \;-\; P(t_{i+1}) - \text{score}(m_{i+1}). \end{aligned}$$

The heuristic (Def. 1) can then be rewritten as follows:

$$\begin{aligned} & h^{\mathcal{M}}_{p,c_{\text{seed}}}(u) \\ & = \min_{\substack{u \preceq_p m_1 \preceq_p \cdots \preceq_p m_l \preceq_p v_t \\ m_i \in \mathcal{M}}} \sum_{0 \le i \le l} \big[P(t_i) - P(t_{i+1}) - \text{score}(m_{i+1})\big] \\ & = P(u) - \max_{\substack{u \preceq_p m_1 \preceq_p \cdots \preceq_p m_l \preceq_p v_t \\ m_i \in \mathcal{M}}} \sum_{0 \le i \le l} \text{score}(m_{i+1}) \\ & = P(u) - S_p(u). \end{aligned}$$

□

Lemma 5 *Layer* $\mathcal{L}_\ell$ $(\ell > 0)$ *is fully determined by the set of those matches* m *for which* $\ell \le S_p(m) < \ell + r$*:*

$$\mathcal{L}_\ell = \{u \mid \exists m \in \mathcal{M} : u \preceq_p m \text{ and } S_p(m) \in [\ell, \ell + r)\}.$$

Proof Take any state $u \in \mathcal{L}_\ell$. Its score $S_p(u) \ge \ell > 0$ implies that there is a non-empty $\preceq_p$-chain $u \preceq_p m_1 \preceq_p m_2 \preceq_p \ldots$ with $\text{score}(m_1) + \text{score}(m_2) + \cdots \ge \ell$. The score of each match is less than r and thus there must be a match m_i so that the subset of the chain starting at m_i has score $S_p(m_i) = \text{score}(m_i) + \text{score}(m_{i+1}) + \cdots +$ in the interval $[\ell, \ell + r)$. This implies that for any u with score $S_p(u) \ge \ell > 0$ there is a match with score in $[\ell, \ell + r)$ succeeding u, as required. □

B.4. Computation of the gap-chaining seed heuristic

In this section we prove (Thm. 5) that we can change the dependency of GCSH on c_{gs} to c_{seed} by introducing a new partial order $\preceq_T$ on the matches. This way, Thm. 4 applies and we can efficiently compute GCSH. Recall that the gap-seed cost is $c_{\text{gs}} = \max(c_{\text{gap}}, c_{\text{seed}})$, and that the gap transformation is:

Definition 4 (Gap transformation) The partial order $\preceq_T$ on states is induced by comparing both coordinates after the *gap transformation*

$$T: \quad \langle i, j \rangle \mapsto (i - j - P\langle i, j \rangle, j - i - P\langle i, j \rangle)$$

The following lemma allows us to determine whether c_{gap} or c_{seed} dominates the cost c_{gs} between two matches, based on the relation $\preceq_T$.

Lemma 6 *Let* u *and* v *be two states with* v *at the start of some seed. Then* $u \preceq_T v$ *if and only if* $c_{\text{gap}}(u, v) \le c_{\text{seed}}(u, v)$*. Furthermore,* $u \preceq_T v$ *implies* $u \preceq v$*.*

Proof Let $u = \langle i, j \rangle$ and $v = \langle i', j' \rangle$. By definition, $u \preceq_T v$ is equivalent to

$$\begin{cases} i{-}j{-}P(u) \le i'{-}j'{-}P(v) \\ j{-}i{-}P(u) \le j'{-}i'{-}P(v) \end{cases} \Leftrightarrow \begin{cases} -\big((i'{-}i) - (j'{-}j)\big) \le P(u) - P(v) \\ \quad (i'{-}i) - (j'{-}j) \;\; \le P(u) - P(v). \end{cases}$$

This simplifies to

$$c_{\text{gap}}(u, v) = |(i' - i) - (j' - j)| \le P(u) - P(v) = c_{\text{seed}}(u, v),$$

where the last equality holds because v is at the start of a seed.

For the second part, $u \preceq_T v$ implies $0 \le c_{\text{gap}}(u, v) \le c_{\text{seed}}(u, v) = P(u) - P(v)$ and hence $P(u) \ge P(v)$. Since v is at the start of a seed, this directly implies $i \le i'$. Since seeds have length $k \ge r$ we have

$$|(i' - i) - (j' - j)| \le P(u) - P(v) = r \cdot |\mathcal{S}_{i \ldots i'}| \le r \cdot (i' - i)/k \le i' - i.$$

This implies $j' - j \ge 0$ and hence $j \le j'$, as required. □

A direct corollary is that for $u \preceq v$ with v at the start of some seed, we have

$$c_{\text{gs}}(u, v) = \begin{cases} c_{\text{seed}}(u, v) & \text{if } u \preceq_T v, \\ c_{\text{gap}}(u, v) & \text{if } u \npreceq_T v. \end{cases} \tag{5}$$

A second corollary is that $\text{start}(m) \preceq_T \text{end}(m)$ for all matches $m \in \mathcal{M}$, since a match from u to v satisfies $c_{\text{gap}}(u, v) < r = c_{\text{seed}}(u, v)$ by definition.

Lemma 7 *When the set of matches* $\mathcal{M}$ *is consistent,* $h^{\mathcal{M}}_{\text{gcs}}(u)$ *can be computed using* $\preceq_T$*-chains only:*

$$h^{\mathcal{M}}_{\text{gcs}}(u) := h^{\mathcal{M}}_{\preceq, c_{\text{gs}}}(u) = \begin{cases} h^{\mathcal{M}}_{\preceq_T, c_{\text{gs}}}(u) & \textit{if } u \preceq_T v_t, \\ c_{\text{gap}}(u, v_t) & \textit{if } u \npreceq_T v_t. \end{cases}$$

Proof We write $h := h^{\mathcal{M}}_{\text{gcs}} = h^{\mathcal{M}}_{\preceq, c_{\text{gs}}}$ (Def. 1) and $h' := h^{\mathcal{M}}_{\preceq_T, c_{\text{gs}}}$.

Case 1: $u \npreceq_T v_t$. Let $u \preceq m_1 \preceq \cdots \preceq m_l \preceq v_t$ be a chain minimizing $h(u)$ in Def. 1, so

$$h(u) = c_{\text{gs}}(u, m_1) + c_{\text{m}}(m_1) + c_{\text{gs}}(m_1, m_2) + \cdots + c_{\text{gs}}(m_l, v_t).$$

By definition, $c_{\text{gs}} \ge c_{\text{gap}}$, and $c_{\text{m}}(m_i) \ge c_{\text{gap}}(\text{start}(m_i), \text{end}(m_i))$, so the weak triangle inequality (Lemma 1) for c_{gap} gives

$$h(u) \ge c_{\text{gap}}(u, m_1) + c_{\text{gap}}(m_1) + \cdots + c_{\text{gap}}(m_l, v_t) \ge c_{\text{gap}}(u, v_t).$$

Since $u \npreceq_T v_t$, the empty chain $u \preceq v_t$ has cost $h(u) \le c_{\text{gs}}(u, v_t) = c_{\text{gap}}(u, v_t)$. Combining the two inequalities, $h(u) = c_{\text{gap}}(u, v_t)$.

Case 2: $u \preceq_T v_t$. First rewrite h and h' recursively as

$$h(u) = \min_{\substack{m \in \mathcal{M} \\ u \preceq m \preceq v_t}} \big(c_{\text{gs}}(u, m) + c_{\text{m}}(m) + \; h(\text{end}(m))\big) \tag{6}$$

$$h'(u) = \min_{\substack{m \in \mathcal{M} \\ u \preceq_T m \preceq_T v_t}} \big(c_{\text{gs}}(u, m) + c_{\text{m}}(m) + h'(\text{end}(m))\big), \tag{7}$$

both with base case $h(v_t) = h'(v_t) = 0$ after eventually taking m_ω. We will show that

$$h(u) = \min_{\substack{m \in \mathcal{M} \\ u \preceq_T m \preceq_T v_t}} \big(c_{\text{gs}}(u, m) + c_{\text{m}}(m) + h(\text{end}(m))\big), \tag{8}$$

which is exactly the recursion for h', so that by induction $h(u) = h'(u)$.

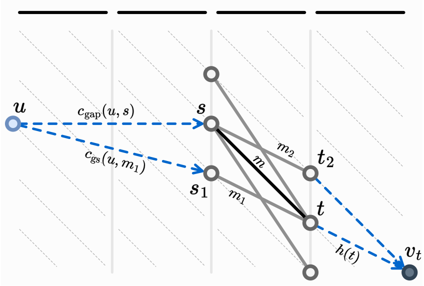

Fig. 7: **Variables in Case 2 of the proof of Lemma 7.** Match m has $\mathrm{score}(m)=2$, so it has adjacent matches m_1 and m_2 (gray) with $\mathrm{score}(m_i)\geq 1$.

By Lemma 6, every $\preceq_T$-chain is a $\preceq$-chain, so $h(u)\leq h'(u)$. To prove $h(u){=}h'(u)$, it remains to show the reverse inequality, $h'(u)\leq h(u)$. To this end, choose a match m that

(priority 0) minimizes $h(u)$ in Eq. (6), and among those, has
(priority 1) maximal $c_{\mathrm{seed}}(u,m)$, and among those, has
(priority 2) minimal $c_{\mathrm{gap}}(u,m)$, and among those, has
(priority 3) minimal $c_{\mathrm{gap}}(m,v_t)$.

We show that $u\preceq_T m$ (in 2.A) and $m\preceq_T v_t$ (in 2.B), which proves Eq. (8).

Part 2.A: $u\preceq_T m$. Let s and t be the begin and end of m (Fig. 7), and let m' be a match minimizing $h(t)$ in Eq. (6) so

$$h(t)=c_{\mathrm{gs}}(t,m')+c_{\mathrm{m}}(m')+h(\mathrm{end}(m')).$$

Since m' comes after t we have $c_{\mathrm{seed}}(u,m')>c_{\mathrm{seed}}(u,m)$ (p.1) and hence m' does not minimize $h(u)$ (p.0):

$$h(u)<c_{\mathrm{gs}}(u,m')+c_{\mathrm{m}}(m')+h(\mathrm{end}(m')).$$

Using the minimality of m, the non-minimality of m', and the triangle inequality we get

$$\begin{aligned}c_{\mathrm{gs}}(u,s)+c_{\mathrm{m}}(m)+h(t)&=h(u)\\&<c_{\mathrm{gs}}(u,m')\qquad\qquad\quad+c_{\mathrm{m}}(m')+h(\mathrm{end}(m'))\\&\leq c_{\mathrm{gs}}(u,t)+c_{\mathrm{gs}}(t,m')+c_{\mathrm{m}}(m')+h(\mathrm{end}(m'))\\&=c_{\mathrm{gs}}(u,t)+h(t)\end{aligned}$$

so we have

$$c_{\mathrm{gs}}(u,m)+c_{\mathrm{m}}(m)<c_{\mathrm{gs}}(u,t). \tag{9}$$

From the triangle inequality for c_{gap}, from $c_{\mathrm{gap}}(u,s)\leq c_{\mathrm{gs}}(u,s)$, and from $c_{\mathrm{gap}}(s,t)\leq c_{\mathrm{m}}(m)$, and from Eq. (9) we obtain

$$c_{\mathrm{gap}}(u,t)\leq c_{\mathrm{gap}}(u,s)+c_{\mathrm{gap}}(s,t)\leq c_{\mathrm{gs}}(u,s)+c_{\mathrm{m}}(m)<c_{\mathrm{gs}}(u,t).$$

This implies $c_{\mathrm{gs}}(u,t)=c_{\mathrm{seed}}(u,t)$ and hence reusing Eq. (9)

$$\begin{aligned}c_{\mathrm{gap}}(u,s)+c_{\mathrm{m}}(m)&\leq c_{\mathrm{gs}}(u,s)+c_{\mathrm{m}}(m)\\&<c_{\mathrm{seed}}(u,t)=c_{\mathrm{seed}}(u,s)+c_{\mathrm{seed}}(s,t).\end{aligned}$$

We have $c_{\mathrm{m}}(m)=c_{\mathrm{seed}}(s,t)-\mathrm{score}(m)$, so the above simplifies to $c_{\mathrm{gap}}(u,s)<c_{\mathrm{seed}}(u,s)+\mathrm{score}(m)$ and since these are integers $c_{\mathrm{gap}}(u,s)\leq c_{\mathrm{seed}}(u,s)+\mathrm{score}(m)-1$.

When $\mathrm{score}(m)=1$, this implies $c_{\mathrm{gap}}(u,s)\leq c_{\mathrm{seed}}(u,s)$ and $u\preceq_T s=\mathrm{start}(m)$ by Lemma 6.

When $\mathrm{score}(m)>1$, suppose that $c_{\mathrm{gap}}(u,s)>c_{\mathrm{seed}}(u,s)\geq 0$. That means that u is either above or below the diagonal of s. Let $s_1=\langle s_i,s_j\pm 1\rangle$ be the state adjacent to s on the same side of this diagonal as u. This state exists since $u\preceq s_1\preceq t$. Then $c_{\mathrm{gap}}(u,s_1)=c_{\mathrm{gap}}(u,s){-}1$, and by consistency of $\mathcal{M}$ there is a match m_1 from s_1 to t with $c_{\mathrm{m}}(m_1)\leq c_{\mathrm{m}}(m){+}1$. Then

$$\begin{aligned}&c_{\mathrm{gs}}(u,s_1)+c_{\mathrm{m}}(m_1)+h(t)\\&\leq c_{\mathrm{gs}}(u,s){-}1+c_{\mathrm{m}}(m){+}1+h(t)=h(u),\end{aligned}$$

showing that m_1 minimizes $h(u)$ (p.0). Also $c_{\mathrm{seed}}(u,m_1)=c_{\mathrm{seed}}(u,m_1)$ (p.1) and $c_{\mathrm{gap}}(u,m_1)<c_{\mathrm{gap}}(u,m)$ (p.2), so that m_1 contradicts the minimality of m. Thus, $c_{\mathrm{gap}}(u,s)>c_{\mathrm{seed}}(u,s)$ is impossible and $u\preceq_T s$.

Part 2.B: $m\preceq_T v_t$. When there is some match m' succeeding m in the chain, we have $m\preceq m'\preceq v_t$ and hence $m\preceq v_t$. Thus, suppose that m is the only match in the chain $u\preceq m\preceq v_t$ minimizing $h(u)$. We repeat the proofs of Part 2.A in the reverse direction to show that $m\preceq_T v_t$.

Since $c_{\mathrm{seed}}(u,m)$ is maximal, $h(u)<c_{\mathrm{gs}}(u,m_\omega)$ and thus

$$h(u)=c_{\mathrm{gs}}(u,s)+c_{\mathrm{m}}(m)+c_{\mathrm{gs}}(m,v_t)<c_{\mathrm{gs}}(u,v_t).$$

By assumption we have $u\preceq_T v_t$ and thus $c_{\mathrm{gs}}(u,v_t)=c_{\mathrm{seed}}(u,v_t)$. This gives

$$\begin{aligned}&c_{\mathrm{seed}}(u,s)+c_{\mathrm{seed}}(s,t){-}\,\mathrm{score}(m)+c_{\mathrm{gap}}(t,v_t)\\&\qquad<c_{\mathrm{seed}}(u,v_t)=c_{\mathrm{seed}}(u,s)+c_{\mathrm{seed}}(s,t)+c_{\mathrm{seed}}(t,v_t).\end{aligned}$$

Cancelling terms we obtain $c_{\mathrm{gap}}(t,v_t)<c_{\mathrm{seed}}(t,v_t)+1$ and since they are integers $c_{\mathrm{gap}}(t,v_t)\leq c_{\mathrm{seed}}(t,v_t)+\mathrm{score}(m){-}1$. When $\mathrm{score}(m)=1$, this implies $t\preceq_T v_t$, as required.

When $\mathrm{score}(m)>1$, suppose that $c_{\mathrm{gap}}(t,v_t)>c_{\mathrm{seed}}(t,v_t)$. Let $t_2=\langle t_i,t_j\pm 1\rangle$ be the state adjacent to t on the same side of the diagonal as v_t. By consistency of $\mathcal{M}$ there is a match m_2 from s to t_2 with $\mathrm{score}(m_2)\geq\mathrm{score}(m){-}1$ and $c_{\mathrm{gap}}(t_2,v_t)=c_{\mathrm{gap}}(t,v_t)-1$. Then m_2 minimizes $h(u)$ (p.0)

$$\begin{aligned}&c_{\mathrm{gs}}(u,s)+\ c_{\mathrm{m}}(m_2)\ +\ c_{\mathrm{gs}}(t_2,v_t)\\&\leq c_{\mathrm{gs}}(u,s)+c_{\mathrm{m}}(m){+}1+c_{\mathrm{gs}}(t,v_t){-}1=h(u),\end{aligned}$$

and furthermore $c_{\mathrm{seed}}(u,m_2)=c_{\mathrm{seed}}(u,m)$ (p.1), $c_{\mathrm{gap}}(u,m_2)=c_{\mathrm{gap}}(u,m)$ (p.2), and $c_{\mathrm{gap}}(m_2,v_t)<c_{\mathrm{gap}}(m,v_t)$ (p.3), contradicting the choice of m, so $c_{\mathrm{gap}}(t,v_t)>c_{\mathrm{seed}}(t,v_t)$ is impossible and $t\preceq_T v_t$. ☐

Theorem 5 *Given a* consistent *set of matches* $\mathcal{M}$*, the gap-chaining seed heuristic can be computed using scores in the transformed domain:*

$$h^{\mathcal{M}}_{\mathrm{gcs}}(u)=\begin{cases}P(u)-S_T(u) & \text{if } u\preceq_T v_t,\\ c_{\mathrm{gap}}(u,v_t) & \text{if } u\not\preceq_T v_t.\end{cases}$$

Proof Write $h:=h^{\mathcal{M}}_{\mathrm{gcs}}$ and $h':=h^{\mathcal{M}}_{\preceq_T,c_{\mathrm{gs}}}$. When $u\not\preceq_T v_t$, $h(u)=c_{\mathrm{gap}}(u,v_t)$ by Lemma 7. Otherwise, when $u{\preceq_T}v_t$, we have $h(u)=h'(u)$. Let $u{\preceq_T}m_1{\preceq_T}\dots{\preceq_T}v_t$ be a $\preceq_T$-chain for h' as in Def. 1. All terms in h' satisfy $\mathrm{end}(m_i)\preceq_T\mathrm{start}(m_{i+1})$, so $c_{\mathrm{gap}}\leq c_{\mathrm{seed}}$ and by Lemma 6 $c_{\mathrm{gs}}(m_i,m_{i+1})=c_{\mathrm{seed}}(m_i,m_{i+1})$. Thus, $h'=h_{\preceq_T,c_{\mathrm{seed}}}$, and $h^{\mathcal{M}}_{\mathrm{gcs}}(u)=P(u)-S_T(u)$ by Thm. 4. ☐

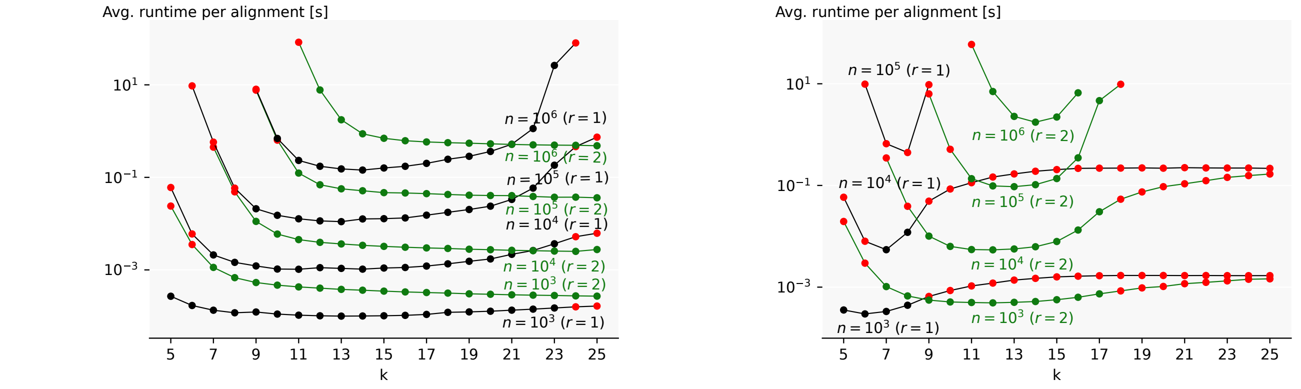

(a) $d = 4\%$ (b) $d = 12\%$

Fig. 8: **Parameter grid search on synthetic data** ($N = 10^7$). Runtime of A*PA with GCSH with DT for varying k and r at $n \in \{10^3, 10^4, 10^5, 10^6\}$ bp and (a) d=4% and (b) d=12%. Black lines indicate r=1 and green lines indicate r=2. Missing datapoints are either due to timing out at $n/N \cdot 1000$ s, or in 2 cases due to exceeding the 32 GiB memory limit. Red dots indicate alignments where k is too small or large (see Sec. C.1).

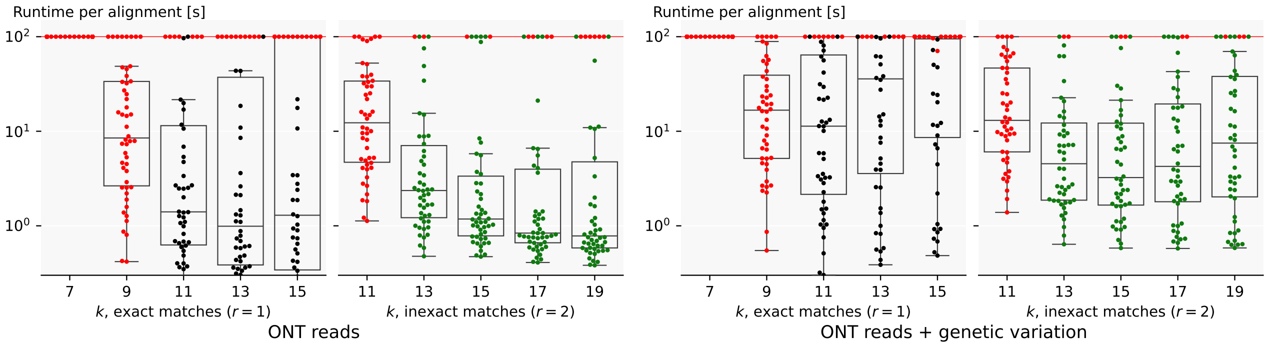

Fig. 9: **Parameter grid search on human data** (logarithmic). Plots show the runtimes on human ONT reads for varying k and r for A*PA with GCSH with DT. ONT reads are without (left) and with (right) genetic variation. Each dot shows the runtime for aligning a single sequence pair (capped at 100 s). Red dots indicate alignments where k is too small or large (see Sec. C.1).

C. Further results

C.1. A*PA parameter grid search

We ran a parameter grid search over various r and k with 5 parallel jobs for both synthetic data and human data.

Synthetic data. Figs. 8a and 8b compare the runtime of A*PA with GCSH with DT for various k for both exact and inexact matches. For low divergence, exact matches are faster since they have a sufficiently large potential and do not require the overhead of finding all inexact matches, while for large divergence, the additional potential of inexact matches is required. In both cases, k=15 is a reasonable choice.

Human data. Fig. 9 shows that r=2 and k=15 minimize the runtime of the third quartile on both datasets. On the ONT reads without genetic variation, choosing a larger k slightly reduces the median runtime, but increases the number of timeouts, making k=15 a reasonable tradeoff.

Bounds on k. The runtime is generally not very sensitive to the exact choice of k as long as it falls within two bounds. First, too many spurious matches are prevented by setting $k \geq \log_4 n$ when r=1 and $k \geq 2 + \log_4 n$ when r=2. For example, n=10^6 would require $k \geq 10$ when r=1 or $k \geq 12$ when r=2. Secondly, the potential $P \approx r \cdot n/k$ of the heuristic should be sufficiently larger than the divergence, which implies k should be a bit less than r/d. So, for d=12% we would choose $k \leq 1/0.12 = 8.3$ when r=1 and $k \leq 2/0.12 = 16.7$ when r=2.

Figs. 8 and 9 show alignments where k is not within these bounds in red, and indeed these alignments are relatively slow.

C.2. Runtime scaling with length

In Fig. 4a we compare our A* heuristics with Edlib and BiWFA in terms of runtime scaling with sequence length n.

C.3. Expanded states and equivalent band

The main benefit of an A* heuristic is a lower number of expanded states, which translates to faster runtime. Instead of evaluating the runtime scaling with length (Fig. 10), we can judge how well a heuristic approximates the edit distance by directly measuring the *equivalent band* (Fig. 11) of each alignment: the number of expanded states divided by sequence length n, or equivalently, the number of expanded states per base pair. The theoretical lower bound is an equivalent band of 1, resulting from expanding only the states on the main diagonal.

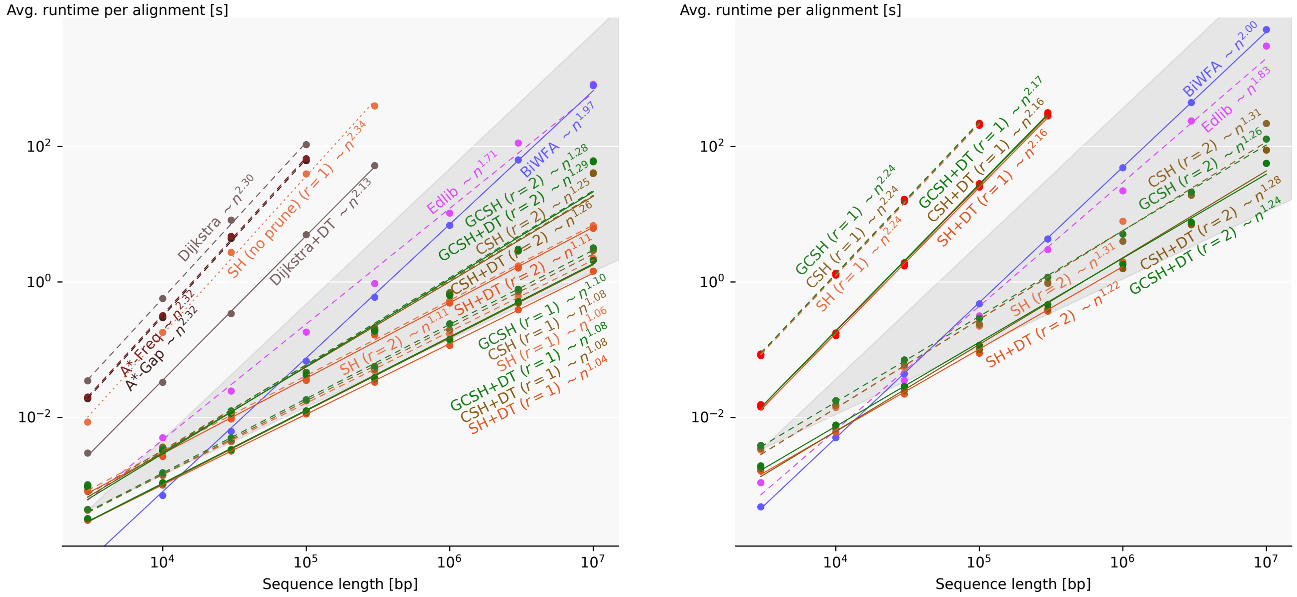

(a) d=4%

(b) d=12%

Fig. 10: **Runtime scaling with sequence length on synthetic data.** Log-log plots comparing our heuristics with EDLIB and BIWFA. The slopes of the bottom (top) of the dark-grey cones correspond to linear (quadratic) growth. The seed length is k=15. Averages are over total N=10^6 bp to N=10^7 bp. SH without pruning is dotted, and variants with DT are solid. For d=12%, red dots show where the heuristic potential is less than the edit distance. Missing data points are due to exceeding the 32 GiB memory limit.

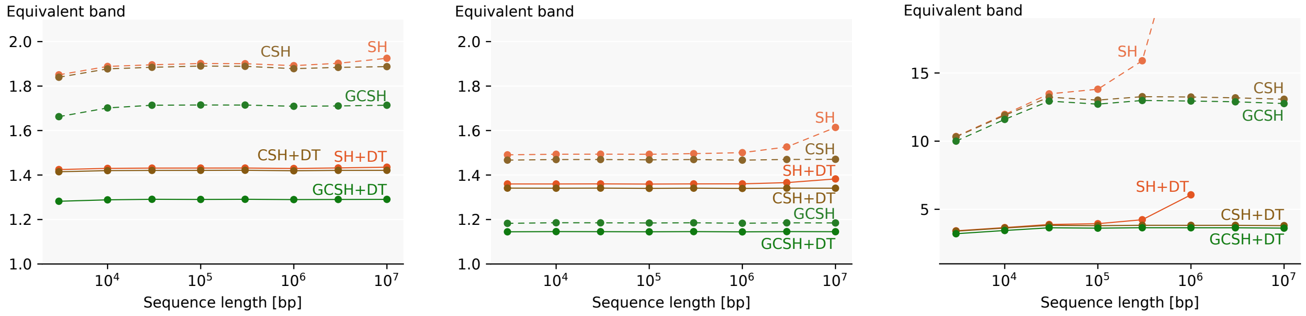

(a) d=4%, exact matches

(b) d=4%, inexact matches

(c) d=12%, inexact matches

Fig. 11: **Equivalent band scaling with sequence length on synthetic data.** (k=15). The equivalent band is the number of expanded states per bp for aligning synthetic sequences. Averages are over total N=10^7 bp.

The equivalent band tends to be constant in n, indicating that the number of expanded states is linear on the given domain. The equivalent band of SH with inexact matches starts to grow around $n \geq 3 \cdot 10^6$ at divergence d=4%, and around $n \geq 3 \cdot 10^5$ at d=12%. Because of the chaining, CSH and GCSH cope with spurious matches and remain constant in equivalent band (i.e. linear expanded states with n). The equivalent band for GCSH is lower than CSH due to better accounting for indels. The DT variants expand fewer states by skipping non-farthest reaching states, also lowering the equivalent band.

C.4. Runtime scaling with divergence: two modes

Figure 12 shows the runtime scaling with divergence for various heuristics. We notice two regimes of operation, depending on whether the heuristic potential P is sufficient to compensate for the edit distance: near-linear in n (constant in d) and quadratic in n (linear in d). The edit distance becomes larger than the potential P around $d = r/k$. For k=15 as in Fig. 12, the threshold is near $d \approx 1/k$=6.7% for exact matches and near $d \approx 2/k$=13.3% for inexact matches. Every error not accounted for by the heuristic triggers a search "to the side", causing A* to explore $O(n)$ additional states. When using DT, only $O(s-P)$ additional farthest reaching states are explored instead, where s is the edit distance. This leads to observed runtimes of $O(n + n \cdot \max(s-P, 0))$ without DT, and $O(n+\max(s-P, 0)^2)$ with DT. These are similar to EDLIB's $O(ns)$ and BIWFA's $O(n + s^2)$, but skipping over the first P errors.

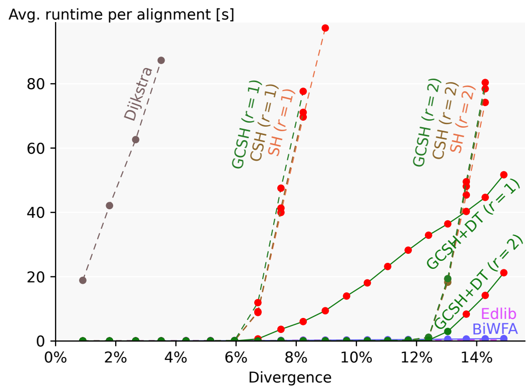

Fig. 12: **Runtime scaling with divergence** TODO LABELS (linear, synthetic, n=10^5, 10^6 bp total, k=15). The figure shows the same results as Fig. 4c, but zoomed out to show show scaling for high d, where runtime of A*PA degrades from constant in d to linear in d. r=1 and r=2 indicate exact and inexact matches, respectively. Red dots show where the heuristic potential is less than the edit distance. Missing datapoints timed out after 100 s.

Dataset	Cnt	Length [kbp]			Divergence [%]			Max gap [kbp]		
		min	mean	max	min	mean	max	min	mean	max
ONT	50	500	594	849	2.7	6.3	18.0	0.02	0.1	1
ONT+gen.var.	48	502	632	1053	4.4	7.4	19.8	0.05	**1.9**	**42**

Table 2. Human datasets statistics. ONT reads only include short gaps, while genetic variation also includes long gaps. **Cnt**: number of sequence pairs. **Max gap**: longest gap in the reconstructed alignment.

C.5. Human data statistic

Statistics on our human datasets are presented in Table 2.

C.6. Memory usage

Table 3 compares memory usage of aligners on synthetic sequences of length n=10^6. Alignments with inexact matches (d≥8%) use significantly more memory than those with exact matches because more k-mer hashes need to be stored to find inexact matches. Table 4 compares memory usage on the human data sets.

Aligner	Memory usage [MB]			
	d=1%	d=4%	d=8%	d=12%
Edlib	2	1	2	2
BiWFA	15	13	14	18
**SH**	50	59	151	480
**CSH**	52	59	151	261
**GCSH**	49	50	151	255
**SH + DT**	49	49	151	150
**CSH + DT**	49	49	151	150
**GCSH + DT**	48	46	152	150

Table 3. Memory usage per algorithm (synthetic data, n=10^6). Exact matches are used when $d \leq 4\%$, and inexact matches when $d \geq 8\%$.

Aligner	ONT reads		+ genetic var.	
	Median	Max	Median	Max
Edlib	2	5	2	6
BiWFA	11	19	15	24
**A*PA (GCSH + DT)**	160	3478	270	6926

Table 4. Memory usage [MB] of aligners on human data. Medians are over all alignments; maximums are over alignments not timing out.

C.7. Runtime profile of A*PA

Figures 13a and 13b compare the time used by stages of A*PA. On synthetic data with exact matches (r=1), the runtime is spread over all parts of the algorithm. When using inexact matches, precomputation takes a significant fraction of the total time and updating contours becomes slower due to the increased number of matches.

On human data, faster alignments spend a large fraction of their time on the precomputation, followed by the updating of contours after pruning matches. Slower alignments on the other hand are limited by the performance of the A* algorithm, and spend a large fraction of time on opening and expanding states, and evaluating the heuristic.

C.8. Quadratic scaling in complex regions

Figure 14 shows the effect of complex regions in the sequences on GCSH (and thus on all our heuristics).

C.9. Linear mode without matches

States are penalized by the number of remaining seeds that cannot be matched. So, curiously, matches are not always needed to direct the A* search to an optimal path. In fact, when each seed contains exactly one mutation seed heuristic scales linearly even though there are no matches, as shown by the artificial example in Fig. 15.

C.10. Comparison of heuristics and techniques

Figure 16 shows the effect of our heuristics and optimizations for aligning complex short sequences. The effect of pruning is most noticeable for CSH and GCSH without DT. GCSH is our most accurate heuristic, so, as expected, it leads to the lowest number of expanded states.

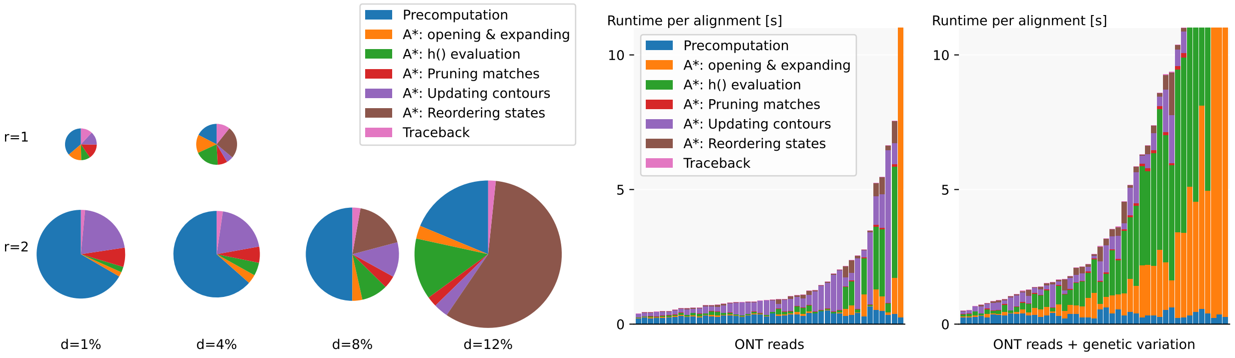

(a) Synthetic data (b) Human data

Fig. 13: **Runtime distributions per stage of A*PA (GCSH with DT)** (stages do not overlap). Stage *A** includes expanding and opening states. *Pruning matches* includes consistency checks. *Updating contours* includes updating of contours after pruning. (a) On synthetic data (n=10^6 bp, N=10^7 bp total, k=15). The circle area is proportional to the total runtime. Figures for r=1 and d≥8% are skipped due to timeouts (100 s). (b) On human data (r=2). Alignments are sorted by total runtime (timeouts not shown).

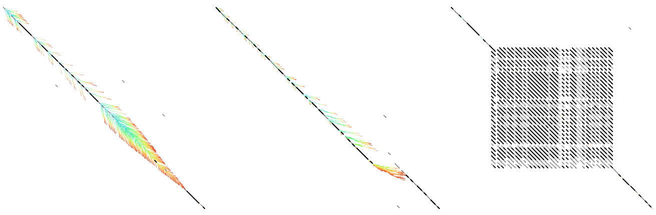

(a) High divergence (b) Long indel (c) Short repeats

Fig. 14: **Quadratic exploration behavior for complex alignments** (GCSH with DT, r=2, k=10, synthetic sequences, n=1000). (a) A highly divergent region, (b) a deletion, and (c) a short repeated pattern inducing a quadratic number of matches. The colour corresponds to the order of expansion, from blue to red.

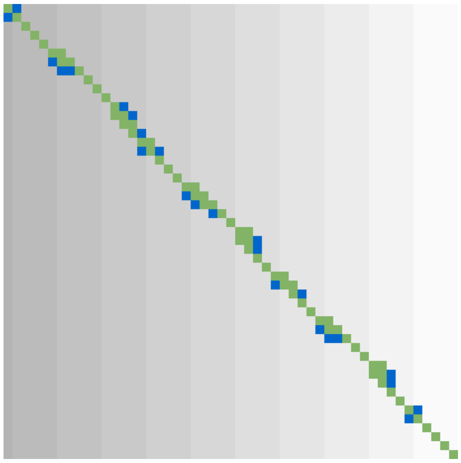

Fig. 15: **Artificial example of A* with seed heuristic with no matches** (n=m=50, r=1, k=5, 80% similarity, 1 mutation per seed alternating substitution, insertion, and deletion). The background colour indicates $h_s(u)$ with higher values darker. Expanded states are green (■), open states blue (■).

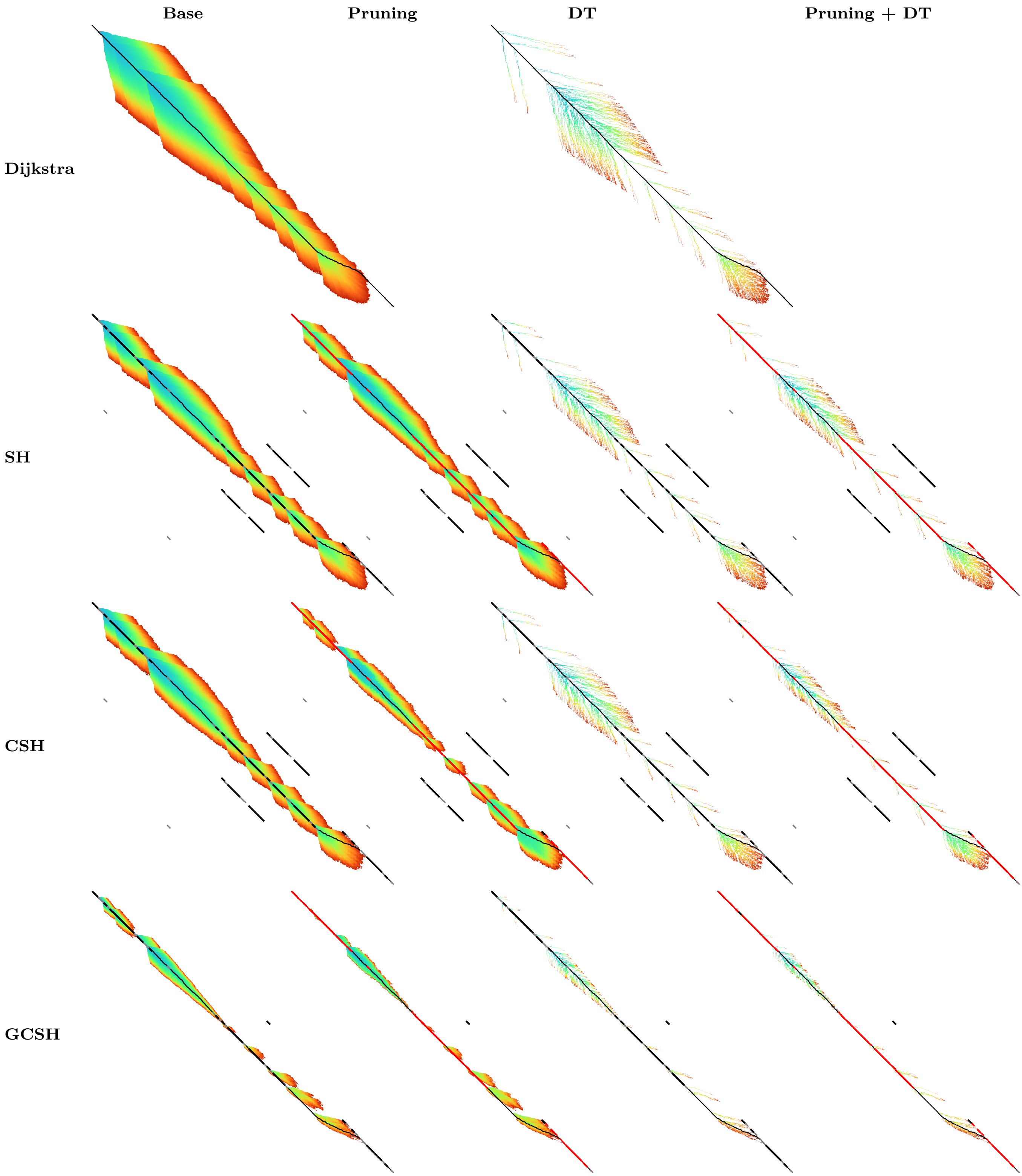

Fig. 16: **Expanded states for various heuristics and techniques, on a sequence containing a noisy region, a repeat, and an indel** (n=1000, d=17.5%). The colour shows the order of expanding, from blue to red. The sequences include a highly divergent region, a repeat, and a gap. Matches are shown as **black diagonals**, with inexact matches in grey and pruned matches in red. The final path is **black**. Dijkstra does not have pruning variants, and Dijkstra with DT is equivalent to WFA. More accurate heuristics reduce the number of expanded states by more effectively punishing repeats (CSH) and gaps (GCSH). Pruning reduces the number of expanded states before the pruned matches, and diagonal transition reduces the density of expanded states in quadratic regions.

D. Notation

Object	Notation
**Sequences**	
Alphabet	$\Sigma = \{\mathtt{A},\mathtt{C},\mathtt{G},\mathtt{T}\}$
Sequences	$A = \overline{a_0 a_1 \dots a_i \dots a_{n-1}} \in \Sigma^*$
	$B = \overline{b_0 b_1 \dots b_j \dots b_{m-1}} \in \Sigma^*$
Substring	$A_{i..i'} = \overline{a_i \dots a_{i'-1}}$
Prefix	$A_{<i} = \overline{a_0 \dots a_{i-1}}$
Suffix	$A_{\geq i} = \overline{a_i \dots a_{n-1}}$
Edit distance	$\mathrm{ed}(A,B)$
Divergence	$d = \mathrm{ed}(A,B)/n$
Error rate	e
**Alignment graph**	
Graph	$G = (V,E)$
Vertices (states)	$u, v \in V = \{\langle i,j\rangle \mid 0 \leq i \leq n, 0 \leq j \leq m\}$
Edges	match/substitution $\langle i,j\rangle \to \langle i{+}1, j{+}1\rangle$
	deletion $\langle i,j\rangle \to \langle i{+}1, j\rangle$
	insertion $\langle i,j\rangle \to \langle i, j{+}1\rangle$
Distance	$\mathrm{d}(u,v)$
Path, shortest path	π, π^*
Cost	$\mathrm{c}_{\mathrm{path}}(\pi)$
**Diagonal transition**	
Farthest-reaching state	$F_{gk} = i{+}j$ on diagonal $k{=}i{-}j$
**A***	
Start and target state	$v_s = \langle 0,0\rangle$, $v_t = \langle n,m\rangle$
Distance from v_s	$g^* = \mathrm{d}(v_s,\cdot)$
Distance to v_t	$h^* = \mathrm{d}(\cdot, v_t)$
Heuristic	h
Best distance from start	g
Estimated distance	$f = g + h$
Admissible heuristic	$h \leq h^*$
Consistent heuristic	$h(u) \leq \mathrm{d}(u,v) + h(v)$
Expanded states	E

Object	Notation
**Seeds and matches**	
Seed length	k
Seed potential	r
Seeds	$s \in \mathcal{S}$, $s_l = A_{lk..lk+k}$
Seeds in suffix	$\mathcal{S}_{\geq i} = \{s_l \in \mathcal{S} \mid lk \geq i\}$
Alignment of seed	π_s
Matches (per seed)	$m \in \mathcal{M}$, $\mathcal{M}_s$, $M = \lvert\mathcal{M}\rvert$
Terminal match	m_ω from v_t to v_t
Cost of match	$0 \leq \mathrm{c_m}(m) < r$
Score of match	$0 < \mathrm{score}(m) = r - \mathrm{c_m}(m) \leq r$
Score of seed	$\mathrm{score}(s) = \max_{m \in \mathcal{M}_s} \mathrm{score}(m)$
**Chains**	
Preceding states	$\langle i,j\rangle \preceq \langle i',j'\rangle$ when $i \leq i'$ and $j \leq j'$
Preceding matches	$m \preceq m'$ when $\mathrm{end}(m) \preceq \mathrm{start}(m')$
	$u \preceq m$ when $u \preceq \mathrm{start}(m)$
Partial order	$u \preceq_p v$ when $p(u) \preceq p(v)$
i-order	$\langle i,j\rangle \preceq_i \langle i',j'\rangle$ when $i \leq i'$
$\preceq_p$-chain	$m_1 \preceq_p \cdots \preceq_p m_l \preceq_p v_t$
**Chaining costs**	
Chaining cost	$\gamma(m,m')$
Gap cost	$c_{\mathrm{gap}}(\langle i,j\rangle, \langle i',j'\rangle) := \lvert(i'{-}i){-}(j'{-}j)\rvert$
Seed cost	$c_{\mathrm{seed}}(u,v) = r \cdot \lvert\mathcal{S}_{u\dots v}\rvert$
Gap-seed cost	$c_{\mathrm{gs}} = \max(c_{\mathrm{gap}}, c_{\mathrm{seed}})$
**Scores**	
Potential	$P\langle i,j\rangle = r \cdot \lvert\mathcal{S}_{\geq i}\rvert$
Chain score	$S_p(m) {=} \max_{m \preceq_p m_1 \preceq_p \cdots \preceq_p v_t} \mathrm{score}(m) + \dots$
	$S_p(u) = \max_{u \preceq_p m \preceq_p v_t} S_p(m)$
Computation	$h_{p,c_{\mathrm{seed}}}(u) = P(u) - S_p(u)$
**Heuristics**	
SH	$h_{\mathrm{s}}(u) = P(u) - S_i(u)$
CSH	$h_{\mathrm{cs}}(u) = P(u) - S_{\preceq}(u)$
GCSH	$h_{\mathrm{gcs}}(u) = \max(c_{\mathrm{gap}}(u,v_t), P(u) - S_T(u))$
	$T : \langle i,j\rangle \mapsto (i{-}j{-}P\langle i,j\rangle, j{-}i{-}P\langle i,j\rangle)$
Pruning heuristic	$\hat{h}^{\mathcal{M}}$
**Layers**	
Layer	$\mathcal{L}_\ell = \{u \mid S_p(u) \geq \ell\}$
Dominant state	$u \in \mathcal{L}_\ell$ s.t. $\{v \in \mathcal{L}_\ell \mid u \preceq v\} = \{u\}$
